# 12-epi-Turpelline, a Novel C20 Diterpene Alkaloid Isolated from Zanba Stir-Fried Tiebangchui

**DOI:** 10.3390/molecules31030479

**Published:** 2026-01-29

**Authors:** Siqi He, Lei Yang, Shilong Meng, Nana Feng, Yue Liu, Yi Zhang, Donglin Chen

**Affiliations:** 1 West China School of Pharmacy, Sichuan University, Chengdu 610041, China; 2023224050086@stu.scu.edu.cn (S.H.); 2023224050092@stu.scu.edu.cn (L.Y.); mengshilong219@126.com (S.M.); 2 State Key Laboratory of Southwestern Chinese Medicine Resources, School of Ethnic Medicine, Chengdu University of Traditional Chinese Medicine, Chengdu 611137, China; fengnana@stu.cdutcm.edu.cn (N.F.); liuyue2@cdutcm.edu.cn (Y.L.)

**Keywords:** Zanba stir-fried Tiebangchui, diterpene alkaloids, inflammation

## Abstract

*Aconitum pendulum* Busch (Tiebangchui), a traditional Chinese medicinal herb, is recognized for its diverse pharmacological properties and for its significant toxicity. The Zanba stir-frying processing method is commonly employed to mitigate toxicity and enhance efficacy; however, the underlying chemical principles remain insufficiently studied. In order to address this issue, a phytochemical investigation was conducted on the diterpenoid alkaloids present in Zanba-processed Tiebangchui. Eleven diterpenoid alkaloids were isolated by means of chromatographic techniques, including silica gel column chromatography. Their structures were elucidated by extensive spectroscopic analysis (single-crystal X-ray diffraction, IR, HR-ESI-MS) and comparison with literature data. The compounds were identified as 12-epi-turpelline (**1**), 12-epi-napelline (**2**), 12-acetyl-napelline (**3**), azitine (**4**), flavaconitine (**5**), nagarutine C (**6**), nagarutine D (**7**), 16-epi-pyroaconine (**8**), spicatine B (**9**), 3-deoxyaconitine (**10**), and napelline (**11**). Notably, compound **1** was characterized as a novel alkaloid. Additionally, compounds **3**–**9** were isolated from this processed material for the first time. These findings provide crucial chemical insights into the detoxification mechanism of Zanba processing. Subsequent pharmacological evaluation revealed that compounds **10** and **11** exhibit notable anti-inflammatory activities. Moreover, given the structural analogy between the novel alkaloid **1** and the active compound **11**, compound **1** is proposed as a promising lead for future structure–activity relationship studies and semi-synthetic modification.

## 1. Introduction

Tiebangchui (TBC) is the accepted nomenclature for the dried rhizomes of two perennial herbaceous species, *Aconitum pendulum* Busch and *Aconitum flavum* Hand.-Mazz., which belong to the Ranunculaceae family [1]. There is extensive documentation relating to the medicinal use of TBC that extends over millennia. The distribution of this substance is predominantly confined to specific high-altitude regions of China, including Tibet, northwestern Yunnan, Qinghai, and western Sichuan. Its first recorded application is in the seminal Tibetan medical treatise, “Four Medical Tantras,” which documents the potent therapeutic properties of this root tuber for treating contusions, fractures, rheumatic lumbago, and frostbite, while also noting its high toxicity [2]. Modern toxicological studies, consistent with records in the “Jing Zhu Materia Medica,” have substantiated these early warnings, identifying significant cardiotoxicity, neurotoxicity, and gastrointestinal toxicity associated with the unprocessed (raw) material [3]. Consequently, a mandatory processing step is required to mitigate these toxic effects before any internal clinical application of TBC.

Modern research has shown that Tiebangchui, a representative toxic medicinal plant in Tibetan medicine, contains mainly alkaloids, flavonoids, and organic acids [4]. Recent studies on this genus have focused on clarifying its chemical composition and developing methods to control its toxicity. It has been established that both the pharmacological activity and toxicity of TBC are primarily attributed to diterpenoid alkaloids [5]. Nevertheless, the marked toxicity of these compounds necessitates a cautious approach to their clinical utilization. Processing (Pao Zhi) is a vital procedure that is employed to reduce toxicity while preserving the therapeutic efficacy of poisonous herbs. As attested by both historical texts and contemporary studies, the application of heat treatment has been identified as a highly efficacious processing strategy for toxic medicinal materials [6]. The processing of TBC follows the principle of “reducing toxicity while preserving efficacy.” Mechanistically, this process promotes the hydrolysis or thermal decomposition of highly toxic diester–diterpenoid alkaloids into significantly less toxic monoester-type alkaloids [7]. The resulting products retain the fundamental pharmacological properties of raw TBC, including anti-inflammatory [8] and analgesic [9] activities.

Zanba is a foodstuff that has been traditionally consumed as a staple food on the Qinghai–Tibet Plateau. It is produced from highland barley (*Hordeum vulgare* L. var. *nudum* Hook. f.). In addition, it is employed as an excipient in the processing of Tibetan medicinal materials [10]. It is used to reduce toxicity while preserving therapeutic efficacy. However, studies on the Zanba stir-frying processing of Tiebangchui (TBC) remain limited. According to traditional Tibetan processing methods [11], TBC is roasted and wrapped in Zanba to decrease its toxicity. This study focused on the Zanba stir-fried processing of TBC. The qualitative and quantitative analysis of the diterpenoid alkaloids present in the processed product was conducted through the utilization of chromatographic separation techniques. A comparison of these results and the chemical profile of raw TBC material was performed. Furthermore, the in vitro cytotoxic and anti-inflammatory activities of the processed product were evaluated. The objective of this study was to elucidate the manner in which Zanba processing affects the “toxicity–efficacy” relationship of TBC. The findings are expected to provide a scientific basis for understanding the mechanism behind this distinctive Tibetan processing method and to help ensure its clinical safety.

Phytochemical analysis of Zanba stir-fried Tiebangchui led to the identification of a new napelline-type C_20_ diterpenoid alkaloid, designated as 12-epi-turpelline (**1**), along with four known C_20_ diterpenoid alkaloids: 12-epi-napelline (**2**), 12-acetyl-napelline (**3**), azitine (**4**), and napelline (**11**). In addition, six C_19_ diterpenoid alkaloids were isolated: flavaconitine (**5**), nagarutine C (**6**), nagarutine D (**7**), 16-epi-pyroaconine (**8**), spicatine B (**9**), and 3-deoxyaconitine (**10**). Among these, compounds **3**–**9** were obtained from processed Tiebangchui for the first time. This study describes the isolation and structural characterization of compounds **1**–**11**, as well as the evaluation of the anti-inflammatory activity of selected compounds using cell-based assays.

## 2. Results and Discussion

### 2.1. Structural Elucidation of 12-epi-Turpelline

Compound **1** was obtained as colorless crystals. It is readily soluble in methanol and dichloromethane but insoluble in petroleum ether. The compound tested positive with Dragendorff’s reagent and showed a characteristic color reaction when treated with 10% sulfuric acid in ethanol. High-resolution ESI-MS analysis identified a protonated molecular ion at *m*/*z* 376.2487 [M+H]⁺ (calcd for C_22_H_34_NO_4^+^_: 376.2488), corresponding to six degrees of unsaturation. The specific optical rotation was determined to be [α]D^25^ = +0.020 (0.003, MeOH). IR spectroscopy was employed, which revealed the presence of hydroxyl (3379 cm^−1^), methyl (1456.3 cm^−1^), and C-N stretching vibration signals (1377 cm^−1^). The ^1^H NMR (400 MHz, CD_3_OD) and ^13^C NMR (100 MHz, CD_3_OD) spectral data are summarized in Table 1.

The ^13^C NMR and DEPT spectra of compound **1** revealed 22 carbon resonances, which were classified as two methyl groups ($\delta_{C}$ 13.63, 26.36), seven methylene groups ($\delta_{C}$ 112.32, 58.62, 52.37, 39.76, 32.51, 31.04, 24.19), nine methine groups ($\delta_{C}$ 77.79, 72.18, 70.22, 69.28, 67.11, 51.38, 45.92, 45.60, 45.35), and four quaternary carbons ($\delta_{C}$ 154.64, 54.31, 52.12, 35.44). The NMR data indicated the absence of methoxy signals but confirmed the presence of a nitrogen-substituted ethyl group ($\delta_{H}$ 1.09, t, *J* = 7.2 Hz, 3H; $\delta_{C}$ 13.63), one exocyclic double bond ($\delta_{H}$ 5.25, d, 1H; 5.10, s, 1H; $\delta_{C}$ 154.64, 112.32), and four oxygenated carbons bearing hydroxyl groups ($\delta_{C}$ 77.79, 72.18, 70.22, 69.28). Comparative analysis of the NMR data for compounds **1** and **2** indicated highly similar structures. The principal differences include the presence of one additional hydroxylated carbon and the absence of one methylene group in compound **1** relative to compound **2**. Consistent with this observation, the molecular mass of compound **1** was found to be 16 Da higher than that of compound **2**, supporting the assignment of an additional oxygen atom in the structure of compound **1** [12].

The combination of ^13^C-NMR and DEPT spectra provides unequivocal evidence that all four hydroxyl-substituted carbon atoms are tertiary carbons. Consequently, the hydroxyl groups are likely to be located at positions C-1, C-2, C-3, C-6, C-11, C-12, C-14, and C-15. The combination of the ^1^H-NMR spectrum and the $\delta_{H}$4.18 signal (dd, *J* = 11.6 Hz, 6.4 Hz) demonstrates a correlation with H-2 ($\delta_{H}$ 1.74) in the ^1^H-^1^H COSY spectrum. Therefore, it can be inferred that the hydroxyl group is located at the C-1 position. In the HMBC spectrum, the correlation between $\delta_{H}$ 4.14 (d, *J* = 2.2 Hz) and C-16 ($\delta_{C}$ 154.64), C-17 ($\delta_{C}$ 112.32), and C-8 ($\delta_{C}$ 52.12) allows for the assignment of this hydroxyl group to C-15 ($\delta_{C}$ 77.79). $\delta_{H}$ 4.03 (dd, *J* = 9.2 Hz, 5.6 Hz) correlates with C-16 ($\delta_{C}$ 154.64) and C-13 ($\delta_{C}$ 45.60), but shows no correlation with C-8 ($\delta_{C}$ 52.12). This finding indicates that the hydroxyl group is attached to C-12 rather than C-14. In the ^1^H-^1^H COSY spectrum, $\delta_{H}$ 4.28 (dd, *J* = 10.8 Hz, 5.6 Hz) demonstrates coupling with H-12 ($\delta_{H}$ 4.03, *J* = 5.6 Hz) and H-9 ($\delta_{H}$ 1.72). In the HMBC spectrum, $\delta_{H}$ 4.28 (dd, *J* = 10.8 Hz, 5.6 Hz) displays coupling with C-8 ($\delta_{C}$ 52.12) and C-13 ($\delta_{C}$ 45.60), thus indicating that this hydroxyl group is attached to C-11 ($\delta_{C}$ 69.28). Further analysis of the ^1^H-^1^H COSY and HMBC spectra of compound **1** lends further support to the hypothesis that the compound has a fully planar structure.

**Figure 1:**
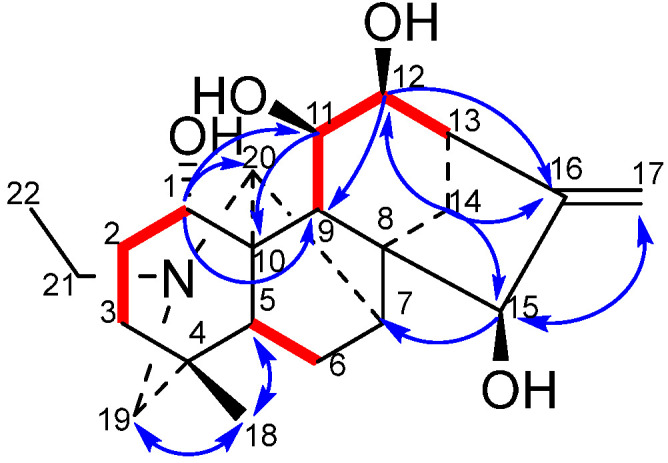
Key ^1^H-^1^H COSY correlations (**—**) and selected HMBC correlations (H**→**C) of compound **1**.

The NOESY diagram provides further confirmation of the stereochemistry of compound **1**. H-1 ($\delta_{H}$ 4.18) is correlated with H-9 ($\delta_{H}$ 1.72), but not with H-21 ($\delta_{H}$ 2.46, 2.61) or H-20 ($\delta_{H}$ 3.42). This finding suggests that H-1 adopts an *α* configuration, while H-11 ($\delta_{H}$ 4.28) and H-14 ($\delta_{H}$ 1.92) demonstrate no correlation with each other. The presence of H-20 ($\delta_{H}$ 3.42) suggests that H-1 adopts an *α* configuration. Furthermore, H-11 ($\delta_{H}$ 4.28) demonstrates a correlation with both H-14 ($\delta_{H}$ 1.91) and H-20 ($\delta_{H}$ 3.42), suggesting the adoption of a *β*-configuration by H-11. Correlation of H-12 ($\delta_{H}$ 4.03) with H-14 ($\delta_{H}$ 1.92) was observed, but not with H-9 ($\delta_{H}$ 1.72). This finding serves to confirm that H-12 adopts a *β*-configuration. H-15 ($\delta_{H}$ 4.14) is correlated with H-7 ($\delta_{H}$ 2.07) and H-14 ($\delta_{H}$ 1.02), thus confirming the hypothesis that H-15 adopts a *β* configuration. This establishes the structure of compound **1**, which is illustrated in Figure 1.

**Figure 2:**
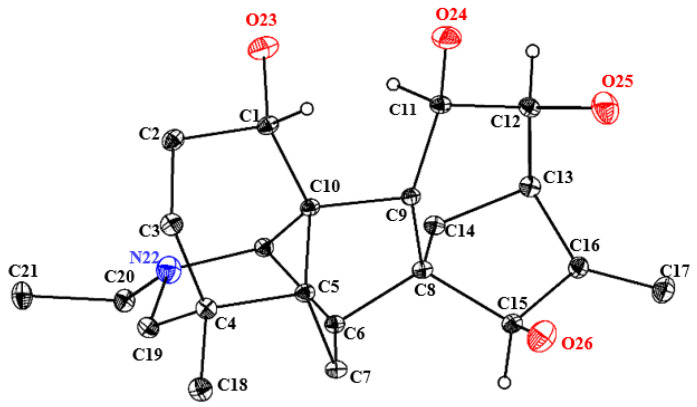
ORTEP drawing of the crystal structure of 12-epi-turpelline.

In view of the uncertainty surrounding the absolute configuration of compound **1**, the observation of its crystal structure in a dichloromethane/methanol solution was indeed fortuitous. Based on its Flack parameter value of 0.13 (18), single-crystal X-ray diffraction analysis (CCDC 2493858) was used to verify its absolute configuration as 1S, 11R, 12S, 15R (Figure 2 and Supplementary Materials Table S1). Thus, compound **1** was definitely established and named 12-epi-turpelline.

### 2.2. Structures of Known Compounds

**Figure 3:**
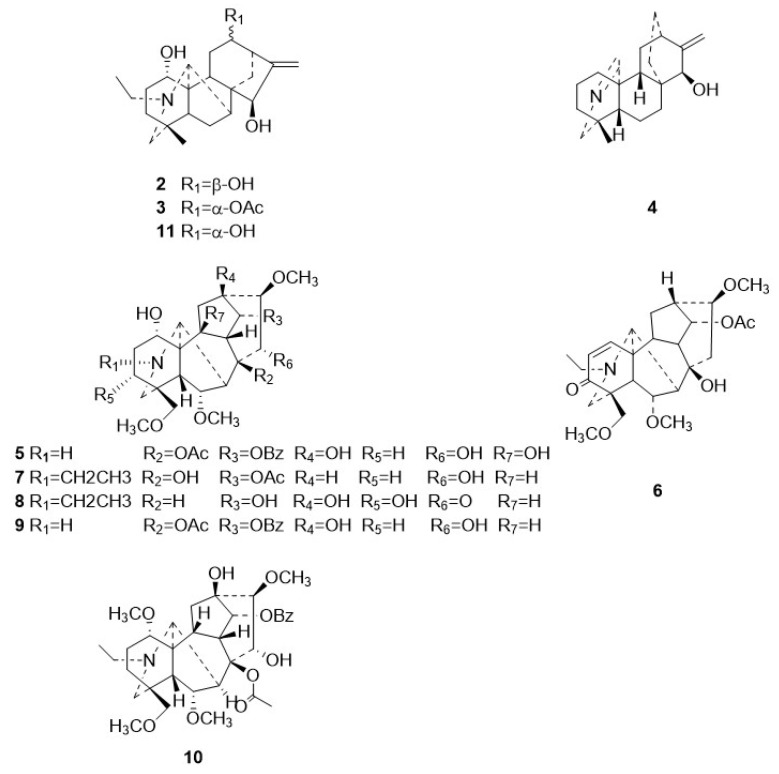
Chemical structures for compounds **2**–**11**.

The following compounds were also isolated from Zanba stir-fried Tiebangchui: 12-epi-napelline (**2**) [13], 12-acetylnapelline (**3**) [12], azitine (**4**) [14,15], flavaconitine (**5**) [12,16], nagarutine C (**6**) [17], nagarutine D (**7**) [17], 16-epi-pyroaconine (**8**) [18], spicatine B (**9**) [19], 3-deoxyaconitine (**10**) [12], and napelline (**11**) [12]. The structures of these compounds were confirmed through a comparison of their ESI-MS and NMR data with those in existing literature reports. Their structural configurations are illustrated in Figure 3.

### 2.3. Evaluation of Anti-Inflammatory Activity

**Figure 4:**
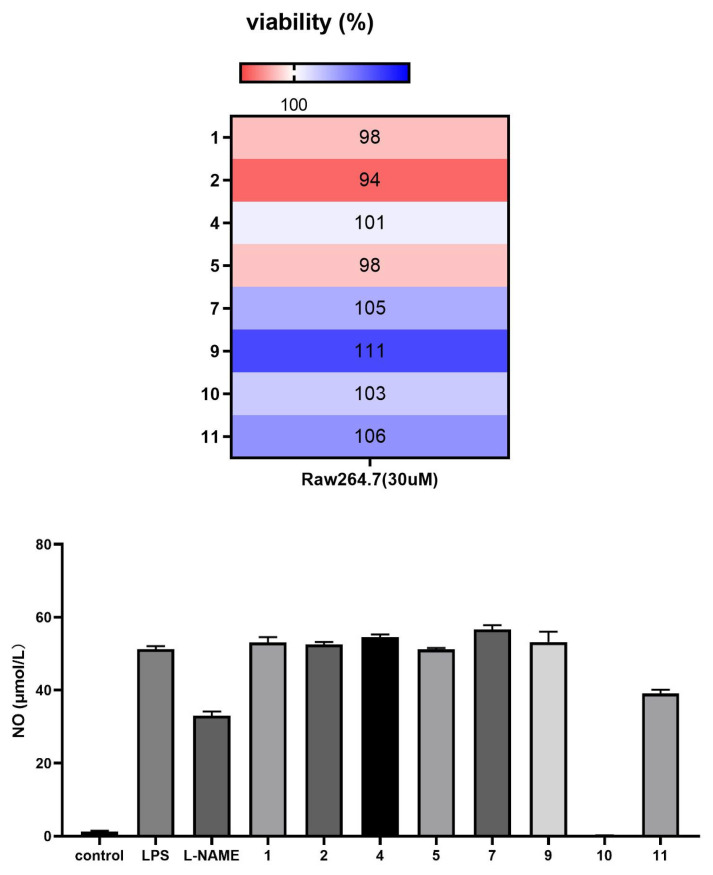
Compounds 1–2, 4–5, 7, and 9–11 exhibit anti-inflammatory activity against LPS-induced inflammation in RAW 264.7 macrophages in vitro.

The anti-inflammatory activity of compounds **1**–**2**, **4**–**5**, **7**, and **9**–**11** against lipopolysaccharide (LPS)-induced inflammation was evaluated in vitro in mouse RAW 264.7 monocyte–macrophages. 3-deoxyaconitine (**10**) and napelline (**11**) demonstrated significant inhibitory activity against LPS-induced NO release in inflammation-related RAW 264.7 cells at a concentration of 30 μM (Figure 4).

## 3. Materials and Methods

### 3.1. General Experimental Procedures

The optical rotation was measured using a Rudolph Research Analytical APV1/6W automatic polarimeter (Rudolph Research Analytical, Hackettstown, NJ, USA). The ultraviolet (UV) spectra of the samples were measured using a UV–vis spectrophotometer (UV-3600, Shimadzu, Kyoto, Japan). The FT-IR spectra of the samples were recorded using a PerkinElmer Spectrum TWO LITA spectrometer (PerkinElmer, Llantrisant, UK) in the range of 4000–400 cm^−1^. 1D- and 2D-NMR spectra were acquired on a Bruker AV-400 NMR instrument (Varian, CA, USA) using TMS as an internal standard in CD_3_OD or CDCl_3_ (^1^H NMR at 400 MHz, ^13^C NMR at 100 MHz). The measurement of MS spectra was conducted utilizing a Water Xevo G2-XS Q-TOF mass spectrometer (Waters, Milford, MA, USA). X-ray crystallographic data were recorded using an APEX-II CCD-equipped single-crystal diffractometer (Bruker D8 Quest, Karlsruhe, Germany) and graphite-monochromated Cu Kα radiation. The column chromatography was performed on silica gel G (100–200, 200–300, 300–400 mesh; Qingdao Marine Chemical Plant, Qingdao, China). All solvents utilized in this study were of analytical grade and were procured from Chengdu Hengxin Reagent Co., Ltd., Chengdu, China, Fractionation was performed using TLC on silica gel GF254 (Qingdao Marine Chemical Plant, Qingdao, China) and visualized by spraying the gel with modified Dragendorff’s reagent. The samples were weighed using an electronic precision balance (JA1003, Lichen Instruments, Shanghai, China).

### 3.2. Plant Material

The experimental Tiebangchui herb was procured from Qinghai in July 2024. Researcher Zhang Yi was the first to identify it as the dried tuber of *Aconitum pendulum* Busch, a plant belonging to the genus *Aconitum* L. within the Ranunculaceae family. The voucher specimen (Specimen No. TBC20210901) is preserved in the Herbarium of the School of Ethnic Medicine, Chendu University of Traditional Chinese Medicine.

### 3.3. Extraction and Isolation

The raw Tiebangchui herb material was processed into slices with a thickness of approximately 2 cm. The Zanba (barley flour) was utilized at a volume three times greater than that of the herb material. The mixture was subjected to stir-frying at 125 °C for a duration of 60 min. This process yielded 20 kg of Zanba stir-fried Tiebangchui herb material, which was then ground and extracted using 95% ethanol at a temperature of approximately −20 °C; the extraction process was repeated five times. The total yield of the extraction process was 5 kg of Zanba stir-fried Tiebangchui extract. The extract was then dispersed in water, and the pH adjusted to 2–3 using 5% HCl. It was extracted twice with ethyl acetate (EA) and the lower aqueous layer was collected. Subsequently, n-butanol was added and the aqueous fraction was then adjusted to a pH greater than 10 using an ammonia solution. Following four rounds of extraction with an equal volume of EA, the EA fraction was concentrated, yielding a total of 78.7 g of alkaloid extract.

**Figure 5:**
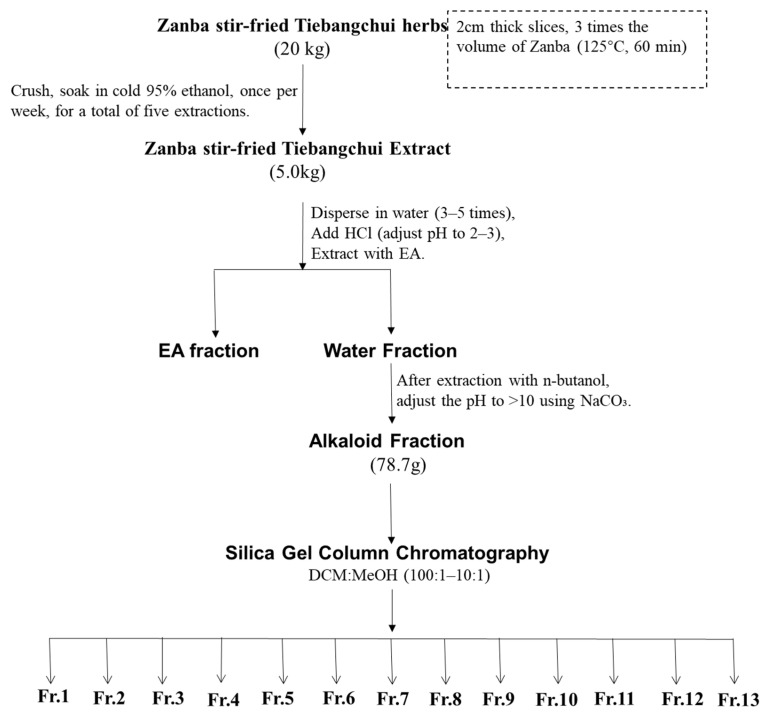
Purification steps for Zanba stir–fried Tiebangchui.

The obtained alkaloids (78.7 g) were then subjected to column chromatography on silica gel (100–200 mesh) using a dichloromethane/methanol system (100:1 to 1:1) for gradient elution, yielding 13 fractions (Fr. 1–Fr. 13) (Figure 5). Fr. 9 (6.4 g) was subjected to a subsequent elution process using a mixture of dichloromethane, methanol, and ammonia (at volumetric ratios of 50:1:1 and 1:1:1). This procedure resulted in the isolation of Fr. 9/1–9/6. Fr. 9/4 was subjected to further elution with a mixture of petroleum ether, acetone, and ammonia water at ratios of 4:1:1 to 1:1:1, yielding compounds **1** (18 mg), **2** (21.5 mg), **3** (20.7 mg), and **10** (38.2 mg). Fr. 7 (3.2 g) was subjected to elution with a dichloromethane/methanol/ammonia solution (100:1:1–90:10:1%), which yielded Fr. 7/1–7/4. Fr. 7/2 was eluted with a petroleum ether, acetone, and ammonia solution (4:1:1–1:1:1%), which yielded compounds **4** (27.1 mg), **5** (40.8 mg), and **6** (27.8 mg). Fr. 12 (5.2 g) was then eluted successively with petroleum ether/acetone/diethylamine (30:5:1–7:2:1), which yielded compounds **7** (9.3 mg), **8** (34.2 mg), **9** (30.8 mg), and **11** (17.3 mg).

### 3.4. Cell Culture and Cytotoxicity Assessment

RAW 264.7 cells were seeded at a density of 1 × 10^6^ cells/mL in a 96-well plate by adding 100 μL of a cell suspension to each well. The experimental groups comprised a blank control group, an LPS model group, an L-NAME positive drug control group [20] (200 μM), and a compound treatment group (30 μM). They were cultured overnight until they had adhered to the plate, and then the medium was replaced and the relevant drugs were administered. Two hours after the administration of the drug treatment, LPS (final concentration: 1 μg/mL) was added to each well, and the cells were then cultured for a further 24 h. Cell supernatants were then collected, and NO release was measured according to the instructions of the Nanjing Jiancheng #A013-2-1 assay kit. Absorbance was detected at 550 nm.

To eliminate the possibility of any cytotoxic interference from compounds **1**–**2**, **4**–**5**, **7**, and **9**–**11**, RAW 264.7 cells were treated with each compound at a concentration of 30 μM for a period of 24 h without LPS. Cell proliferation was measured using the CCK-8 assay.

## 4. Conclusions

In summary, one new diterpenoid alkaloid (12-epi-turpelline, **1**) and ten known compounds (**2**–**11**) were isolated from Zanba stir-fried Tiebangchui. Their structures were determined through comprehensive spectroscopic analysis. The structure of the new compound 1 was unambiguously confirmed by single-crystal X-ray diffraction analysis. This C_20_ diterpenoid alkaloid features an azabicyclo [3.3.1] nonane system (rings A/E), a bicyclo [2.2.1] heptane unit (rings B/F), and an azabicyclo [3.2.1] octane moiety (rings C/D), which contains three fully substituted quaternary carbons at C-4, C-8, and C-10. Cellular activity assays demonstrated that compounds **10** and **11** exhibit significant anti-inflammatory effects in mouse macrophage models. The new alkaloid **1**, which shows structural similarity with the active compound **11**, represents a valuable natural lead compound. Further structural modification and optimization of **1** are warranted to develop more potent anti-inflammatory analogues.

## Figures and Tables

**Table 1:** ^1^H (400 MHz) and ^13^C (100 MHz) NMR data of compound **1** in CD_3_OD.

No.	$\delta_{C}$	$\delta_{H}$ (*J*, Hz)
1	72.18t	4.18 (dd, 11.6, 6.4)
2	31.04d	1.74, 2.49
3	39.76d	1.62, 1.36
4	35.44q	
5	51.38t	1.35
6	24.19d	1.33, 2.32
7	45.92t	2.07
8	52.12q	
9	45.35t	1.72 (10.8)
10	54.31q	
11	69.28t	4.28 (dd, 10.8, 5.6)
12	70.22t	4.03 (dd, 9.2, 5.6)
13	45.60t	2.86 (9.2)
14	32.51d	1.02, 1.92
15	77.79t	4.14 (d, 2.2)
16	154.64q	
17	112.32d	5.10, 5.25 (d, 2.2)
18	26.36s	0.75
19	58.62d	2.30, 2.57
20	67.11t	3.42
21	52.37d	2.46, 2.61
22	13.63s	1.09 (t, 7.2)

## Data Availability

The data supporting the findings of this article are available from the corresponding author, S.H., upon reasonable request.

## Supplementary Materials

The following supporting information can be downloaded at: https://www.mdpi.com/article/10.3390/molecules31030479/s1, Figures S1–S8: Spectral data (HR-ESI-MS, IR, ^1^H NMR, ^13^C NMR and DEPT, ^1^H-^1^HCOSY, HSQC, HMBC, and NOESY) for compound **1**; Table S1: X-ray crystallographic data for compound **1**; Figures S9–S28: Spectral data (^1^H and ^13^C NMR) for compounds **2**–**11**.

## Abbreviations

The following abbreviations are used in this manuscript:

IR	infrared
HR-ESI-MS	high-resolution electrospray ionization mass spectroscopy
NMR	nuclear magnetic resonance
