# Supplementary material for: 12-epi-Turpelline, a Novel C20 Diterpene Alkaloid Isolated from Zanba Stir-Fried Tiebangchui
Source: Molecules. 2026 Jan 29;31(3):479. doi: 10.3390/molecules31030479 (PMC12899062; doi:10.3390/molecules31030479)
Supplement: Supplementary file 1 [file molecules-31-00479-s001.zip › molecules-4088640-supplementary.pdf]

Supporting Information for

12-epi-turpelline, A novel C20 diterpene alkaloid isolated from Zanba stir-fried Tiebangchui

Siqi He [1], Lei Yang [1], Shilong Meng [1], Nana Feng [2], Yue Liu [2], Yi Zhang[2,*] and Donglin Chen [1,*]

[1] West China School of Pharmacy, Sichuan University, Chengdu 610041, China; 2023224050086@stu.scu.edu.cn(S.H.); 2023224050092@stu.scu.edu.cn (L.Y.); mengshilong219@126.com(S.M.)

[2] State Key Laboratory of Southwestern Chinese Medicine Resources, School of Ethnic Medicine, Chengdu University of Traditional Chinese Medicine, Chengdu 611137, China; fengnana@stu.cdutcm.edu.cn(N.F.); liuyue2@cdutcm.edu.cn(Y.L.)

* Correspondence: zhangyi@cdutcm.edu.cn(Y.Z.);chendl@scu.edu.cn(D.C.)

Table of Content

Figure S1. The HRESIMS spectrum of compound **1**....3
Figure S2. IR spectrum of compound **1**. 3
Figure S3. ^{1}H NMR spectrum of compound 1 in CD_3OD 4
Figure S4. ^{13}C NMR and DEPT 135 spectrum of compound **1** in CD_3OD 4
Figure S5. ^{1}H-^{1}H COSY spectrum of compound **1** in CD_3OD 5
Figure S6. HSQC spectrum of compound **1** in CD_3OD....5
Figure S7. HMBC spectrum of compound **1** in CD_3OD....6
Figure S8. NOESY spectrum of compound **1** in CD_3OD 6
Table S1. X-ray crystallographic data for compound **1**....7
Figure S9. ^{1}H NMR (400 MHz) spectrum of compound **2** in $CDCl_3$ 8
Figure S10. ^{13}C NMR (101 MHz) spectrum of compound **2** in $CDCl_3$ 8
Figure S11. ^{1}H NMR (400 MHz) spectrum of compound **3** in $CDCl_3$ 9
Figure S12. ^{13}C NMR (101 MHz) spectrum of compound **3** in $CDCl_3$ 9
Figure S13. ^{1}H NMR (400 MHz) spectrum of compound **4** in $CDCl_3$ 10
Figure S14. ^{13}C NMR (101 MHz) spectrum of compound **4** in $CDCl_3$ 10
Figure S15. ^{1}H NMR (400 MHz) spectrum of compound **5** in $CDCl_3$ 11
Figure S16. ^{13}C NMR (101 MHz) spectrum of compound **5** in $CDCl_3$ 11
Figure S17. ^{1}H NMR (400 MHz) spectrum of compound **6** in $CDCl_3$ 12
Figure S18. ^{13}C NMR (101 MHz) spectrum of compound **6** in $CDCl_3$ 12
Figure S19. ^{1}H NMR (400 MHz) spectrum of compound **7** in $CDCl_3$ 13
Figure S20. ^{13}C NMR (101 MHz) spectrum of compound **7** in $CDCl_3$ 13
Figure S21. ^{1}H NMR (400 MHz) spectrum of compound **8** in $CDCl_3$ 14
Figure S22. ^{13}C NMR (101 MHz) spectrum of compound **8** in $CDCl_3$ 14
Figure S23. ^{1}H NMR (400 MHz) spectrum of compound **9** in $CDCl_3$ 15
Figure S24. ^{13}C NMR (101 MHz) spectrum of compound **9** in $CDCl_3$ 15
Figure S25. ^{1}H NMR (400 MHz) spectrum of compound **10** in $CDCl_3$ 16
Figure S26. ^{13}C NMR (101 MHz) spectrum of compound **10** in $CDCl_3$ 16
Figure S27. ^{1}H NMR (400 MHz) spectrum of compound **11** in $CDCl_3$ 17
Figure S28. ^{13}C NMR (101 MHz) spectrum of compound **11** in $CDCl_3$ 17

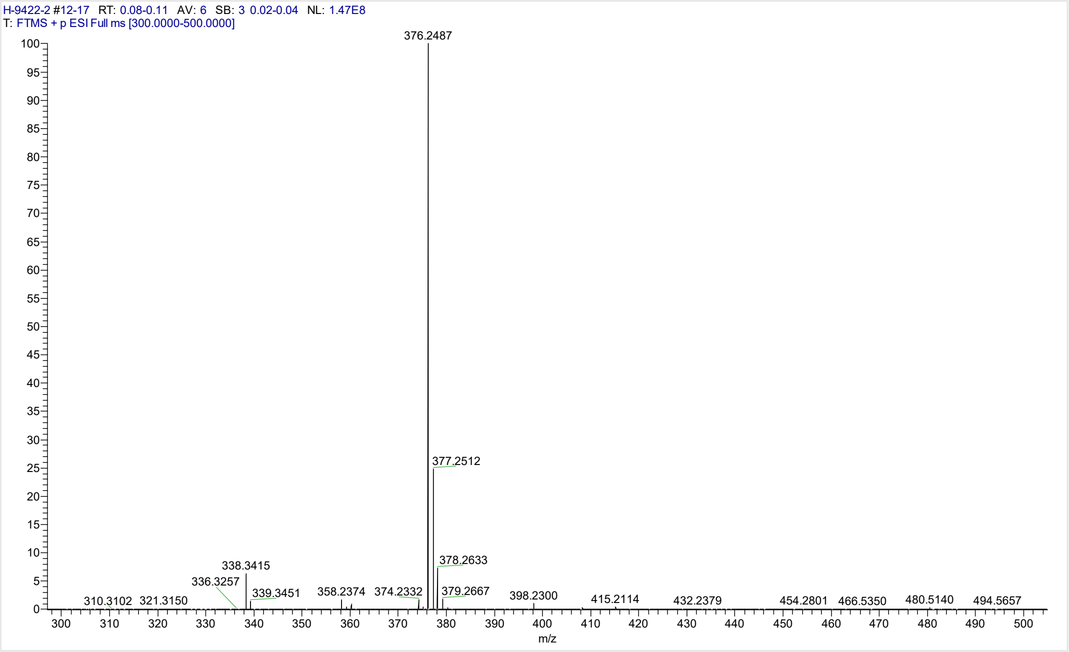

Figure S1. The HRESIMS spectrum of compound **1**.

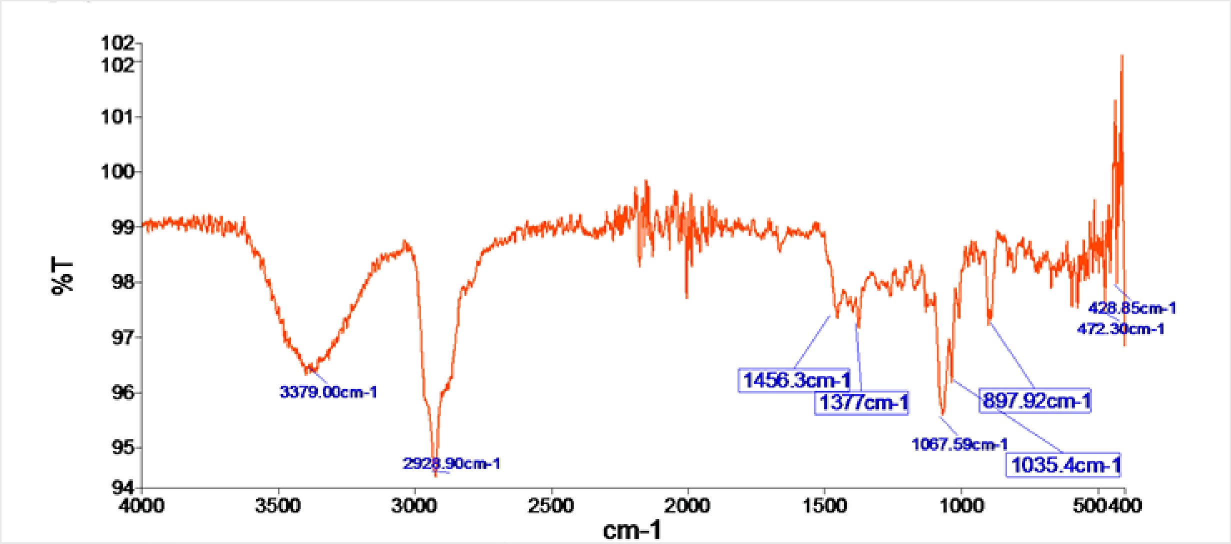

Figure S2. IR spectrum of compound **1**.

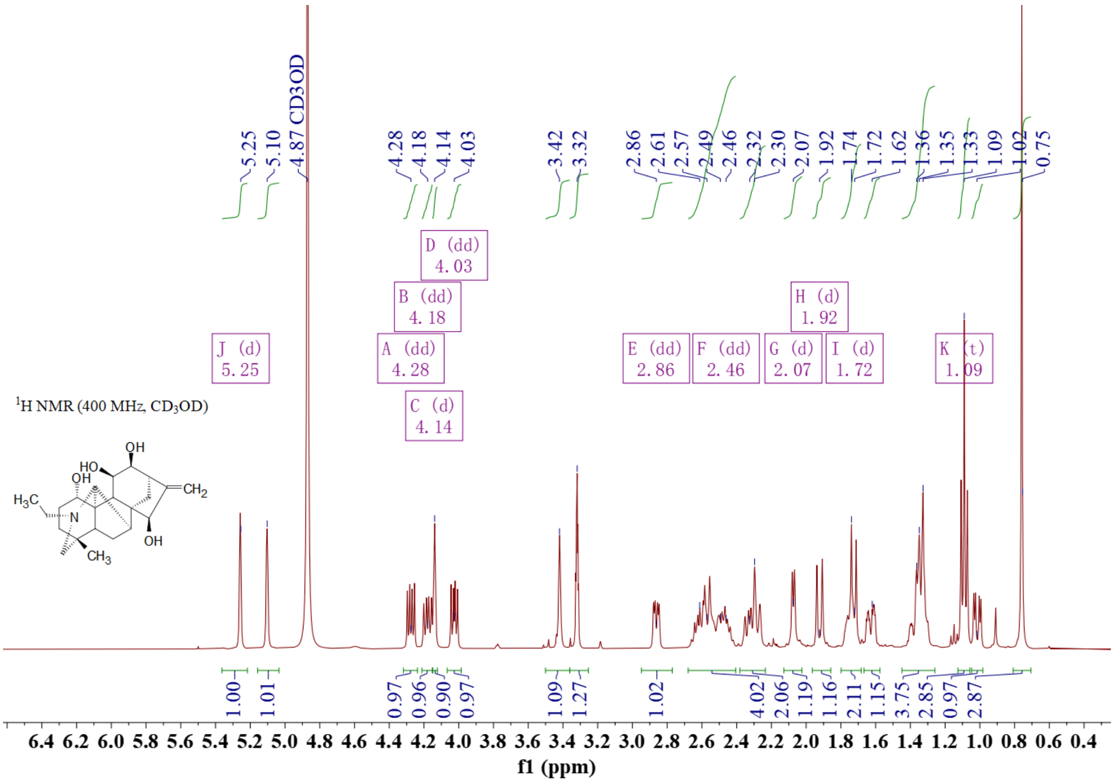

Figure S3. ^{1}H NMR spectrum of compound 1 in CD_3OD

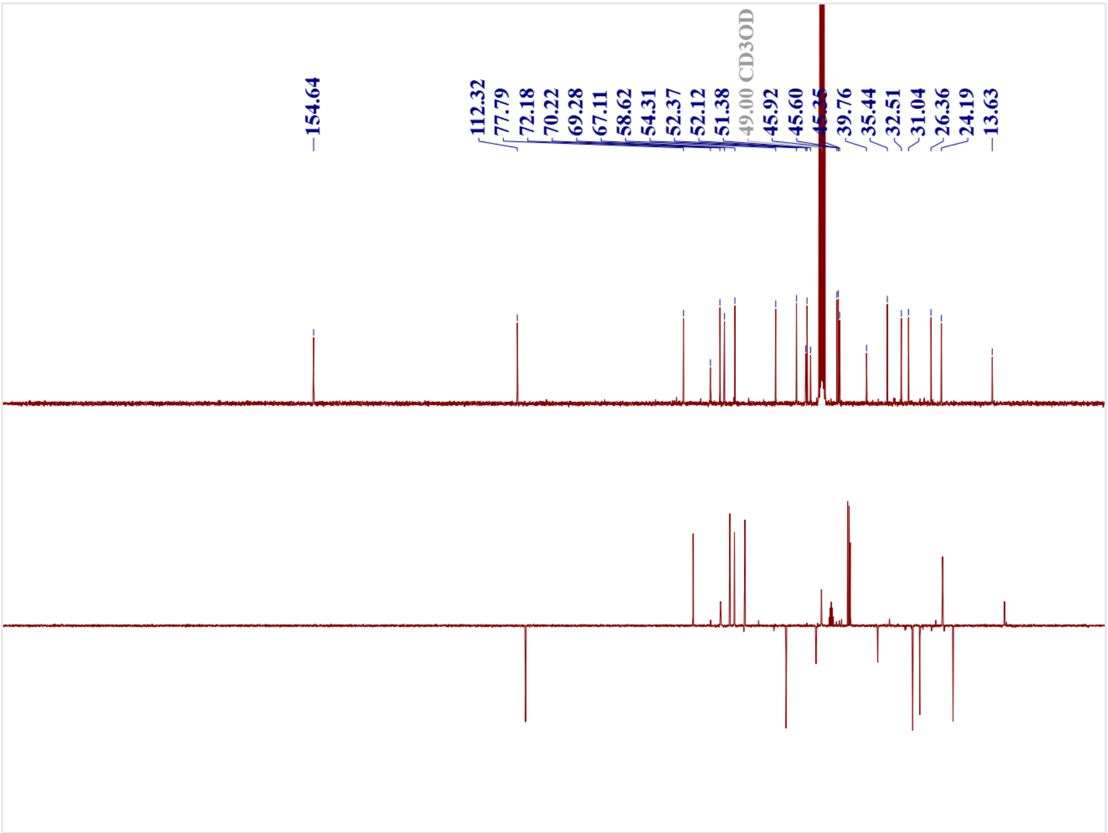

Figure S4. ^{13}C NMR and DEPT 135 spectrum of compound **1** in CD_3OD

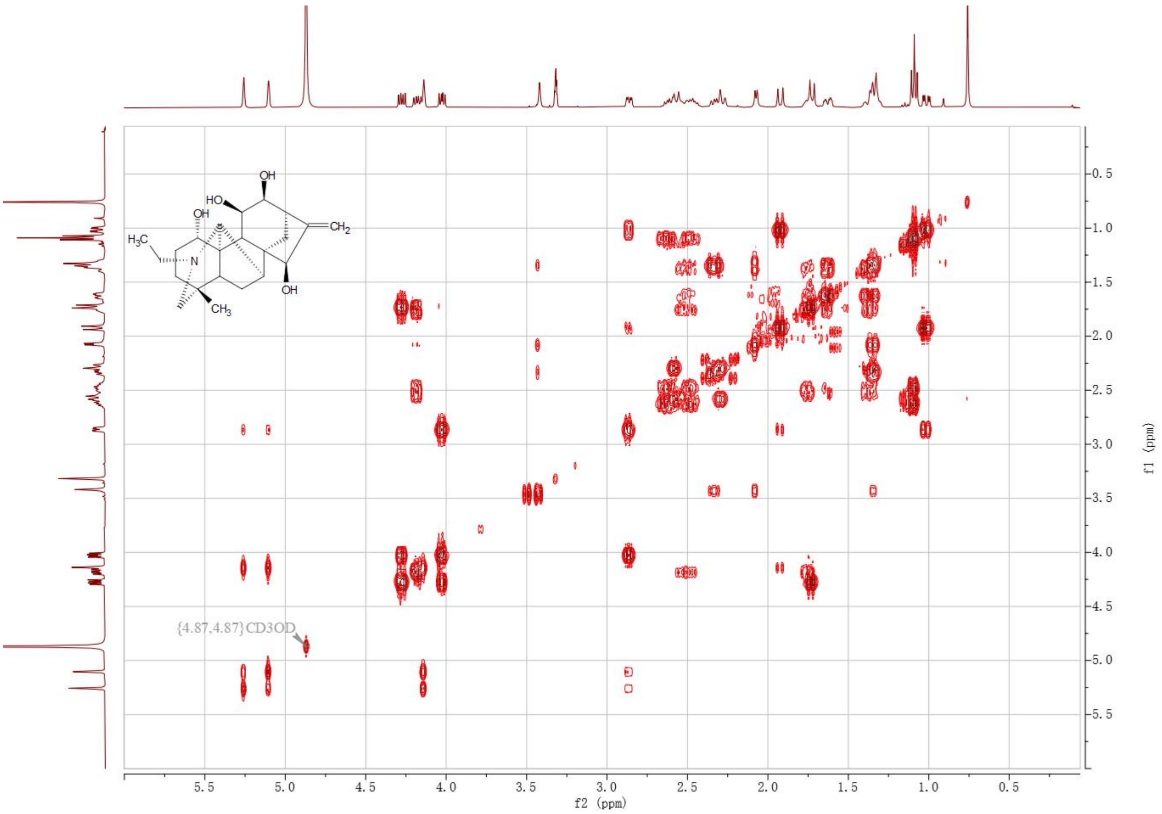

Figure S5. ¹H-¹H COSY spectrum of compound **1** in CD_3OD

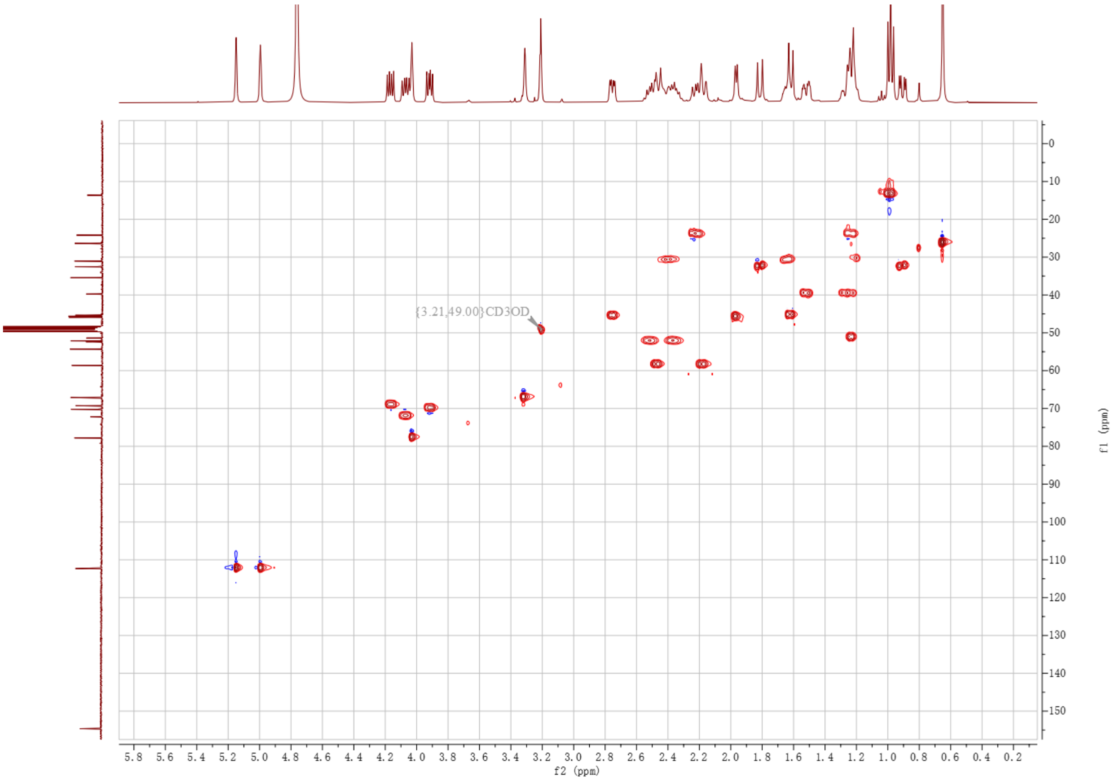

Figure S6. HSQC spectrum of compound **1** in CD_3OD

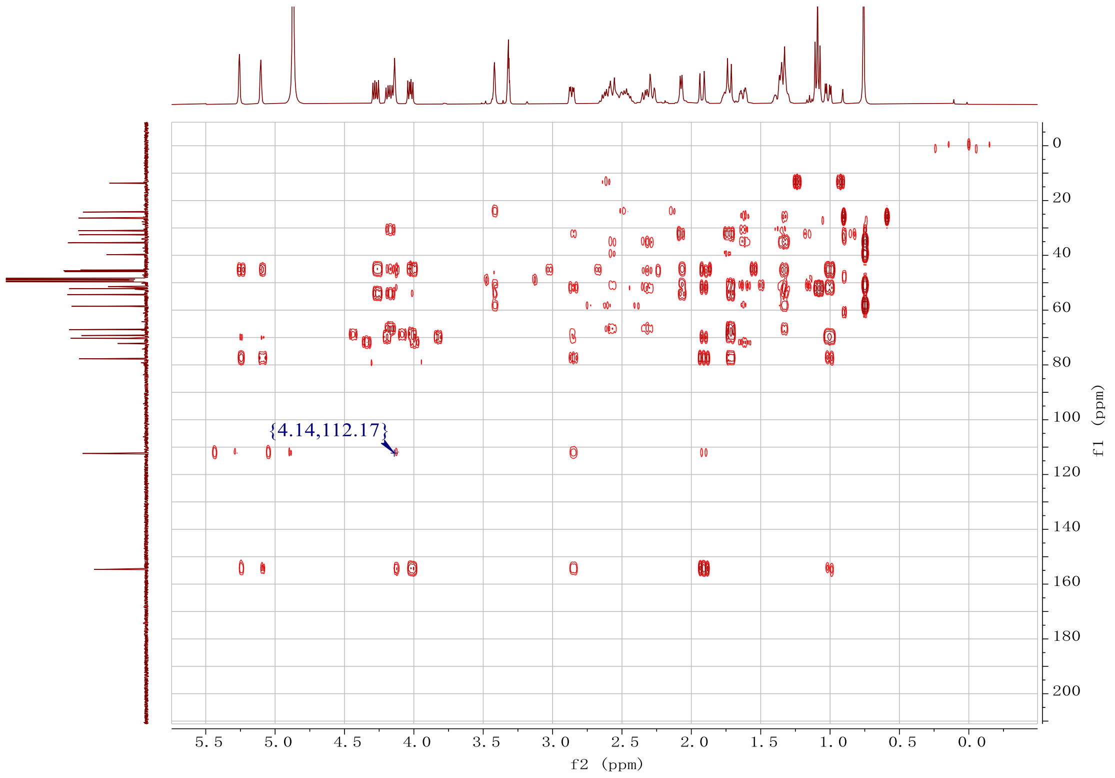

Figure S7. HMBC spectrum of compound **1** in CD_3OD

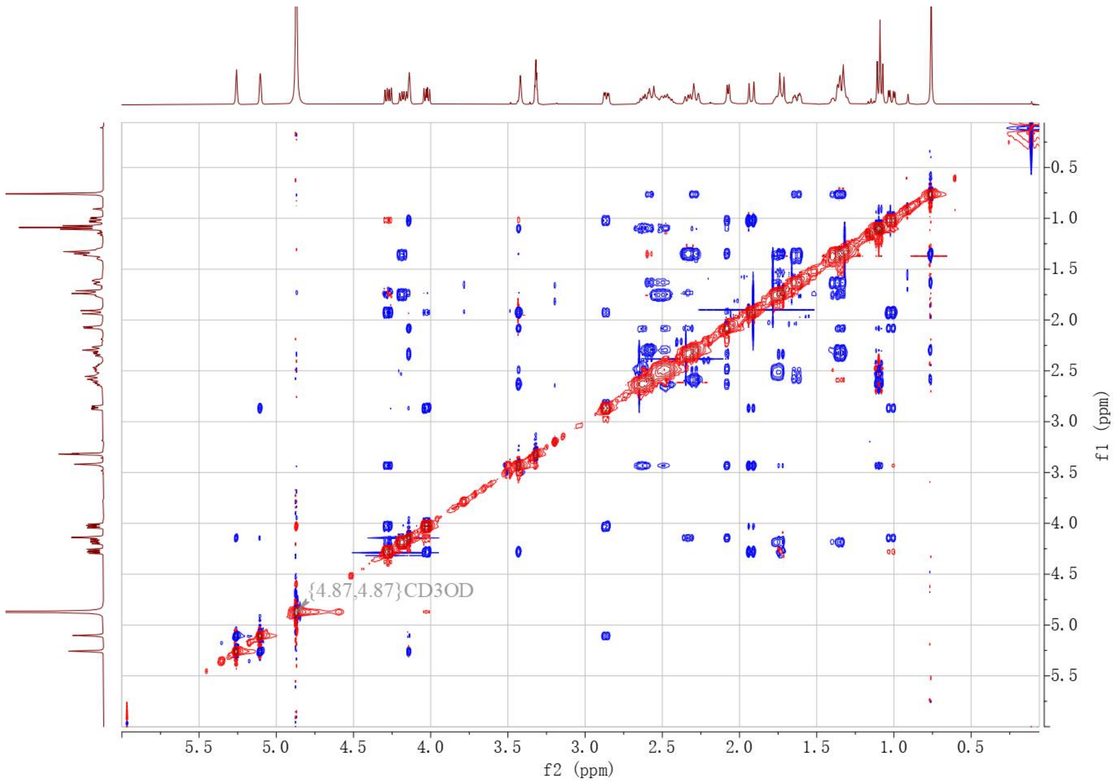

Figure S8. NOESY spectrum of compound **1** in CD_3OD

Table S1. X-ray crystallographic data for compound **1**.

Formula weight	742.92
Temperature/K	293(2)
2Crystal system	tetragonal
Space group	P 43 21 2
a/Å	13.0079(7)
b/Å	13.0079(7)
c/Å	47.370(3)
α/°	90
β/°	90
γ/°	90
Volume/Å3	8015.3(10)
Z	8
ρ_{calc}g/cm3	1.231
μ/mm^{-1}	0.676
F(000)	3200
Radiation	Cu Kα (λ = 1.54178)
2Θ range for data collection/°	3.52 to 68.31°
Index ranges	-13<=h<=13, -15<=k<=15, -57<=l<=57
Reflections collected	68195
Independent reflections	7348 [R_{int} = 0.1218, R_{sigma} = 0.0506]
Data/restraints/parameters	7348/2/491
Goodness-of-fit on F^2	1.083
Final R indexes [I>=2σ (I)]	R_1 = 0.0711, wR_2 = 0.1654
Final R indexes [all data]	R_1 = 0.0978, wR_2 = 0.1891
Largest diff. peak/hole / e Å^{-3}	0.30/-0.26
Flack parameter	0.13(18)

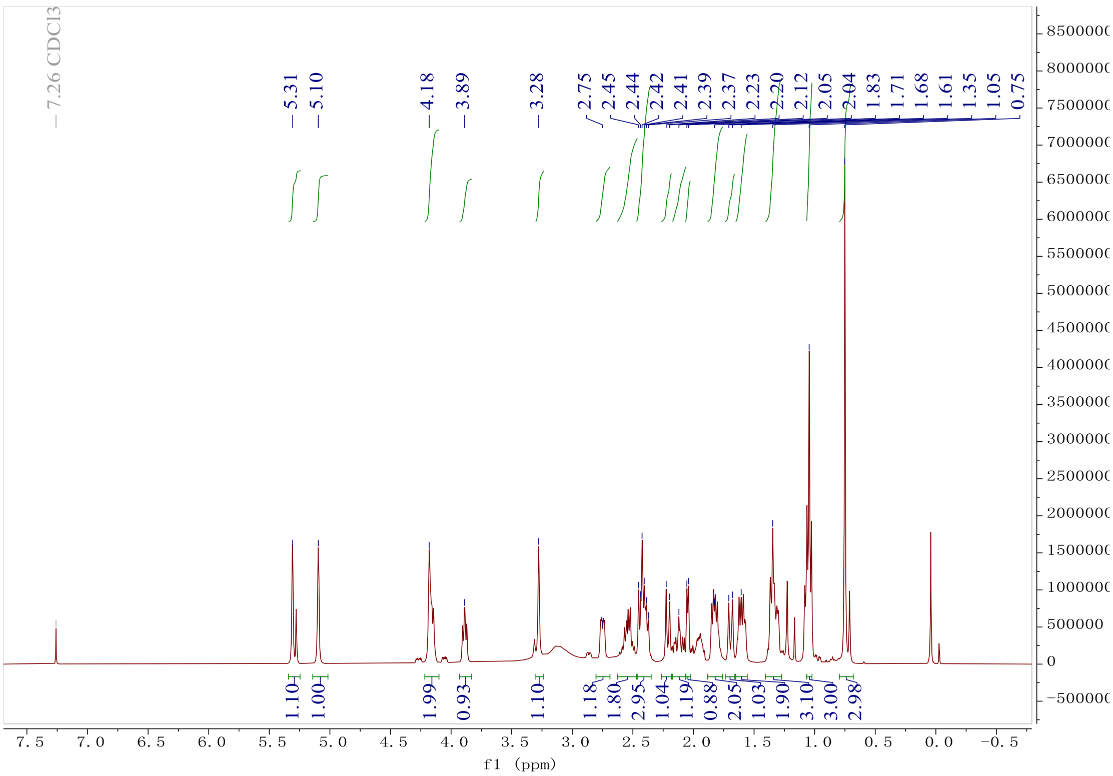

Figure S9. ^{1}H NMR (400 MHz) spectrum of compound **2** in $CDCl_3$

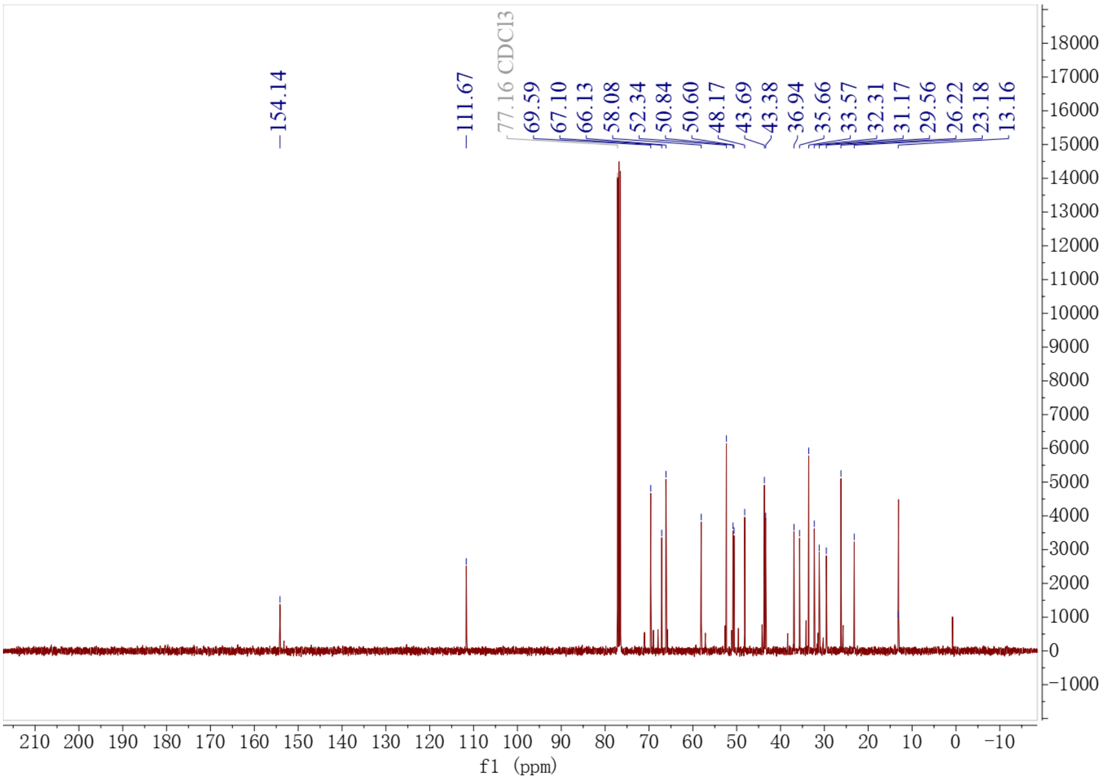

Figure S10. ^{13}C NMR (101 MHz) spectrum of compound **2** in $CDCl_3$

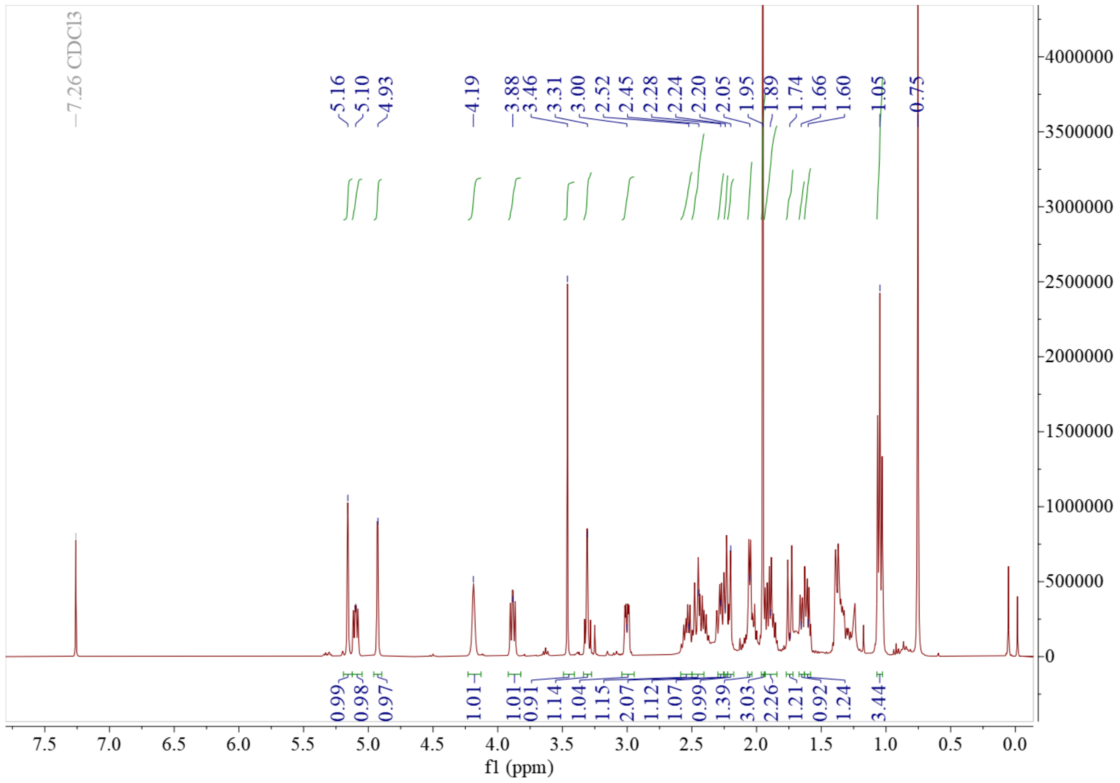

Figure S11. ^{1}H NMR (400 MHz) spectrum of compound **3** in $CDCl_3$

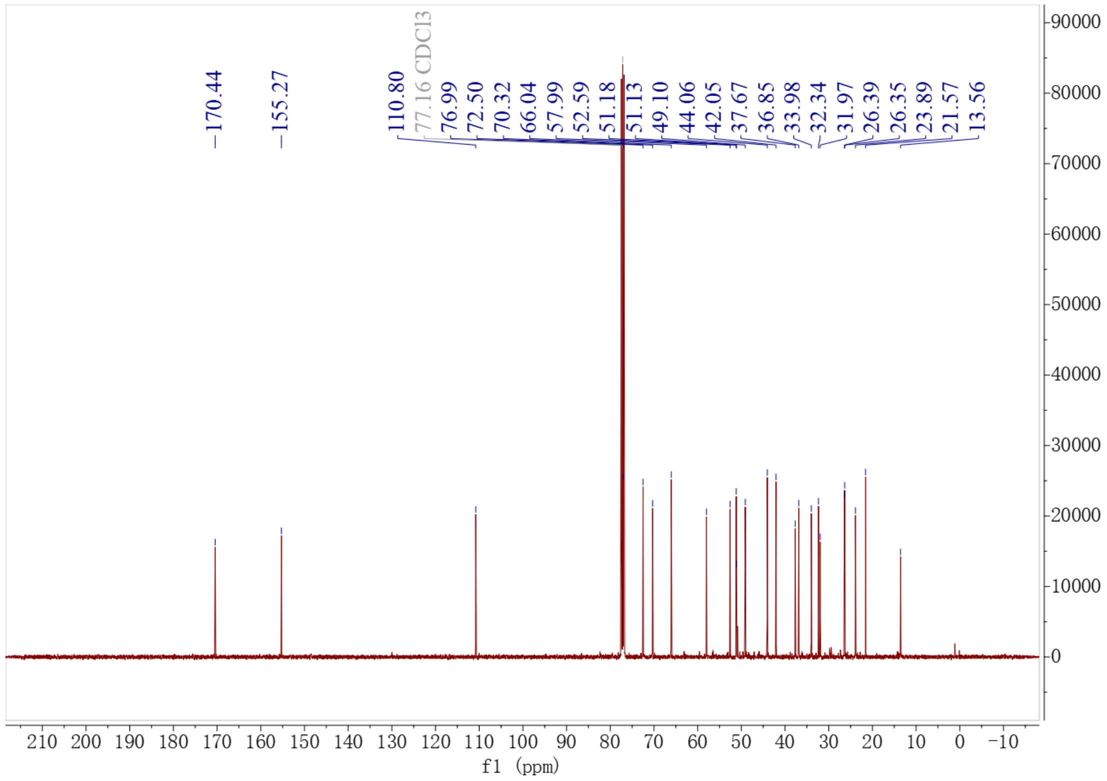

Figure S12. ^{13}C NMR (101 MHz) spectrum of compound **3** in $CDCl_3$

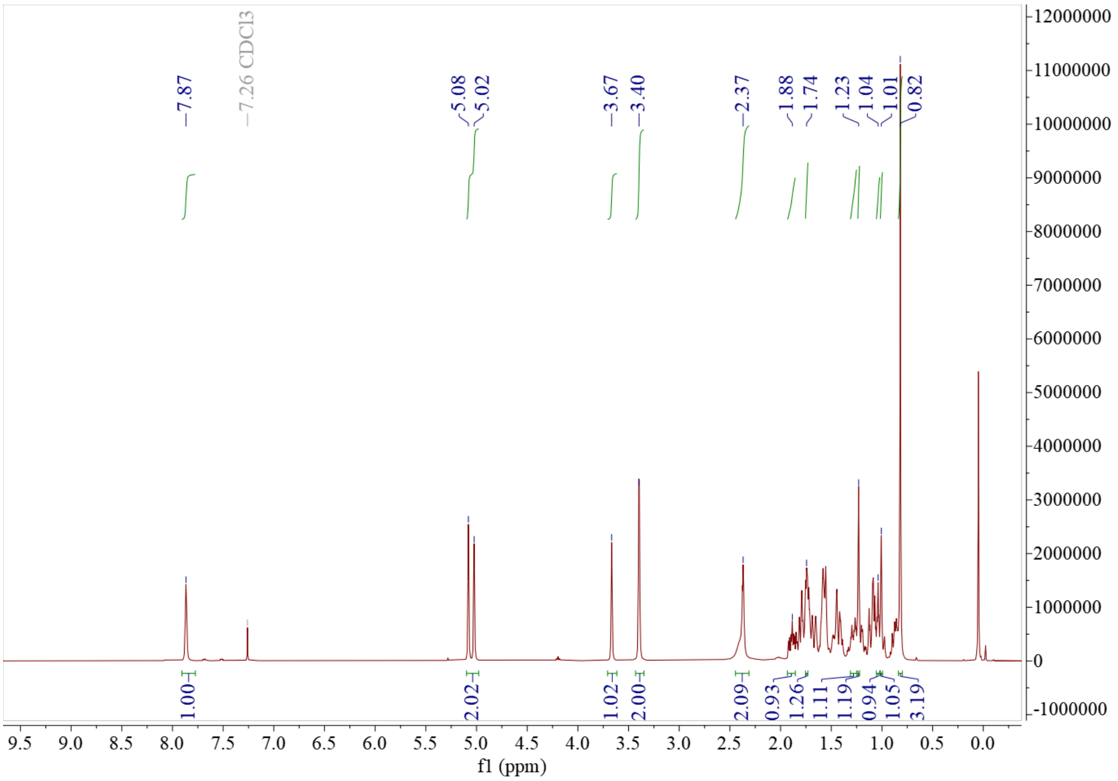

Figure S13. ^{1}H NMR (400 MHz) spectrum of compound **4** in $CDCl_3$

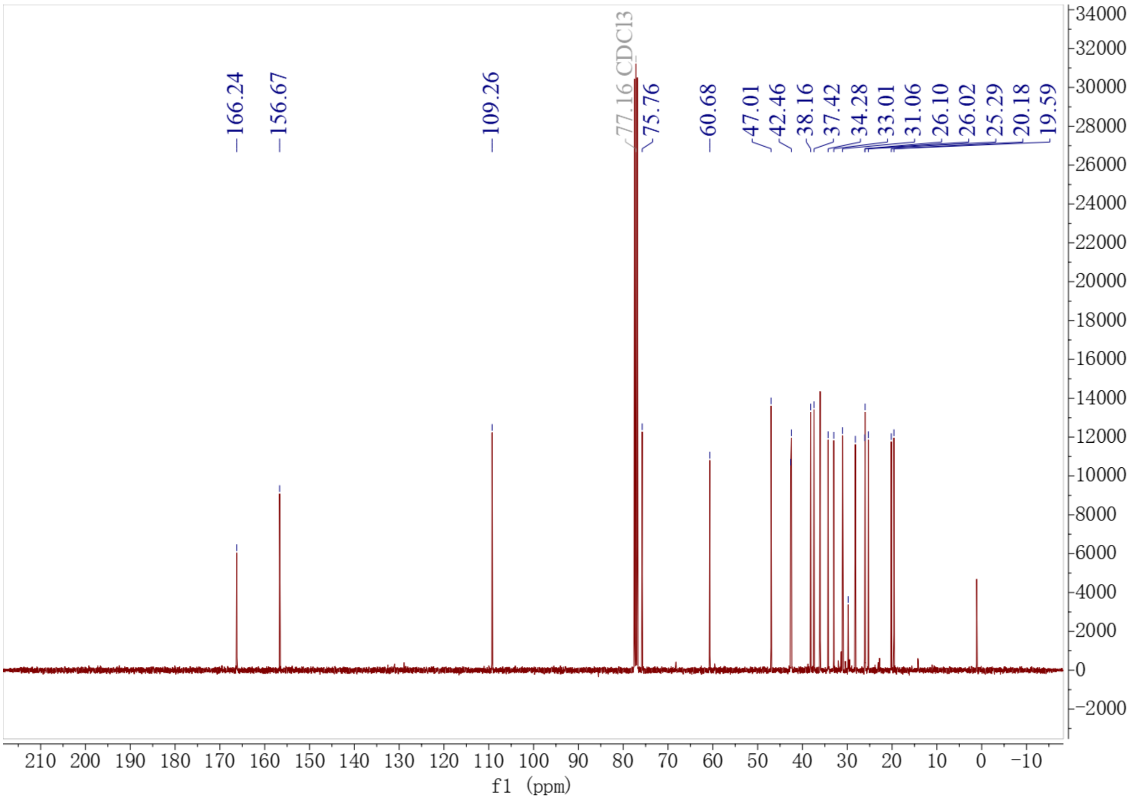

Figure S14. ^{13}C NMR (101 MHz) spectrum of compound **4** in $CDCl_3$

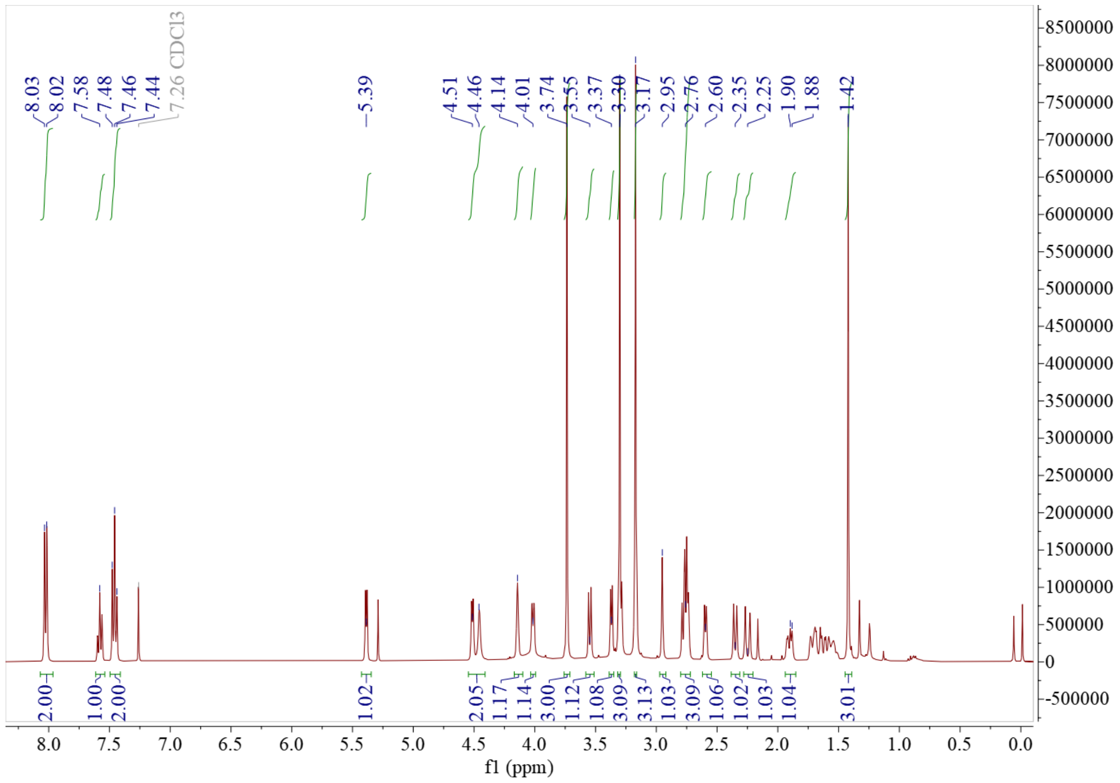

Figure S15. ^{1}H NMR (400 MHz) spectrum of compound **5** in $CDCl_3$

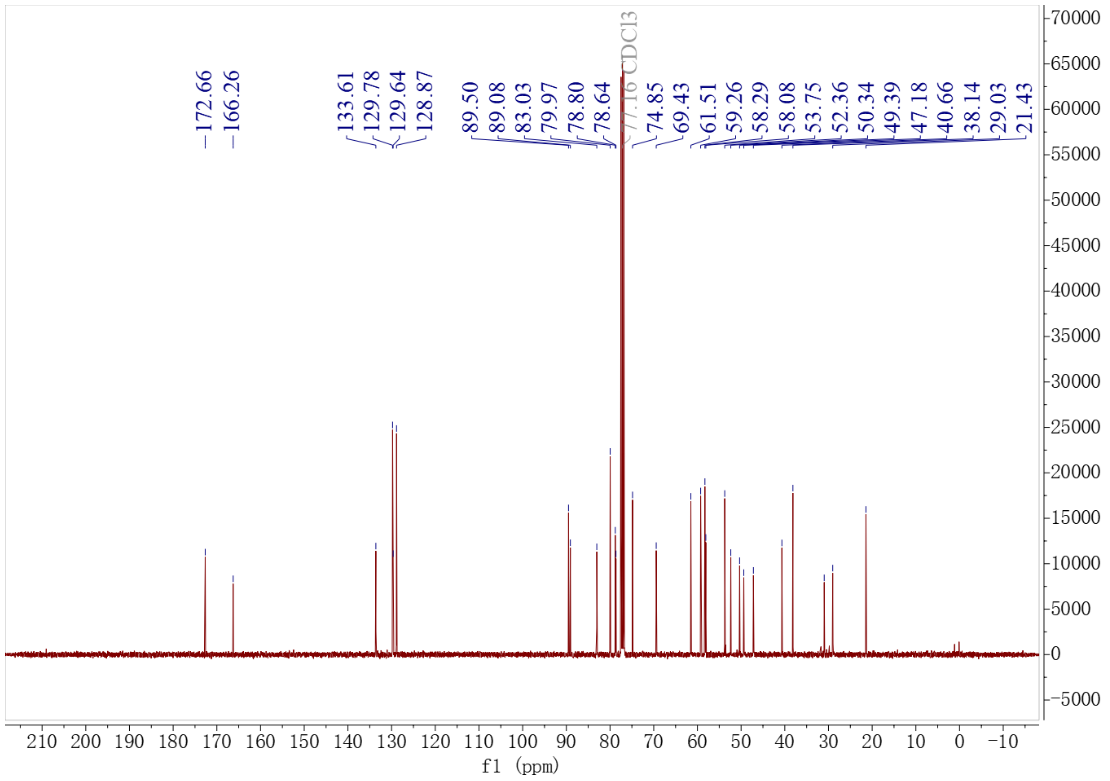

Figure S16. ^{13}C NMR (101 MHz) spectrum of compound **5** in $CDCl_3$

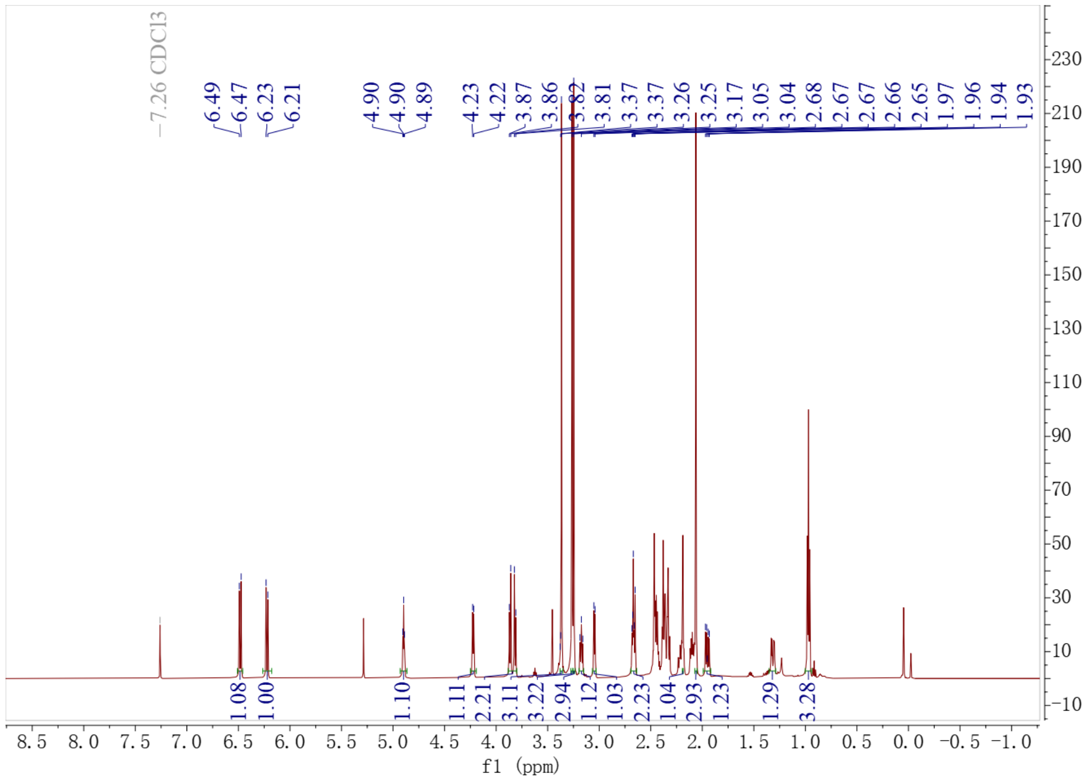

Figure S17. ^{1}H NMR (400 MHz) spectrum of compound **6** in $CDCl_3$

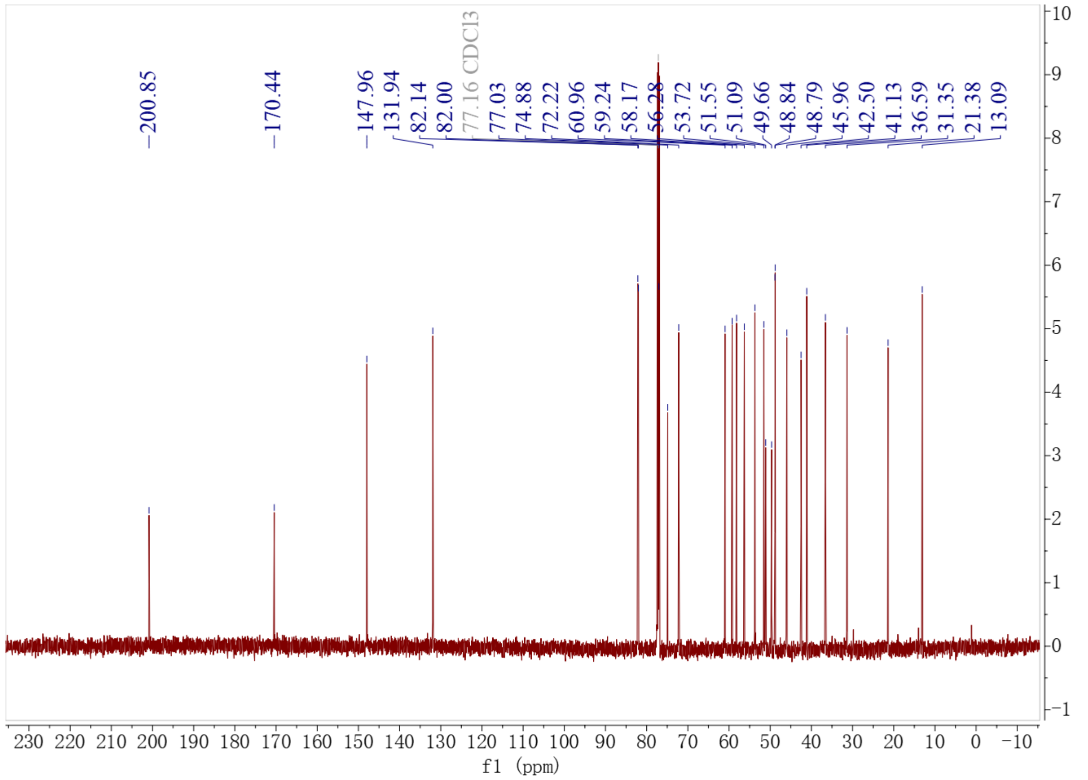

Figure S18. ^{13}C NMR (101 MHz) spectrum of compound **6** in $CDCl_3$

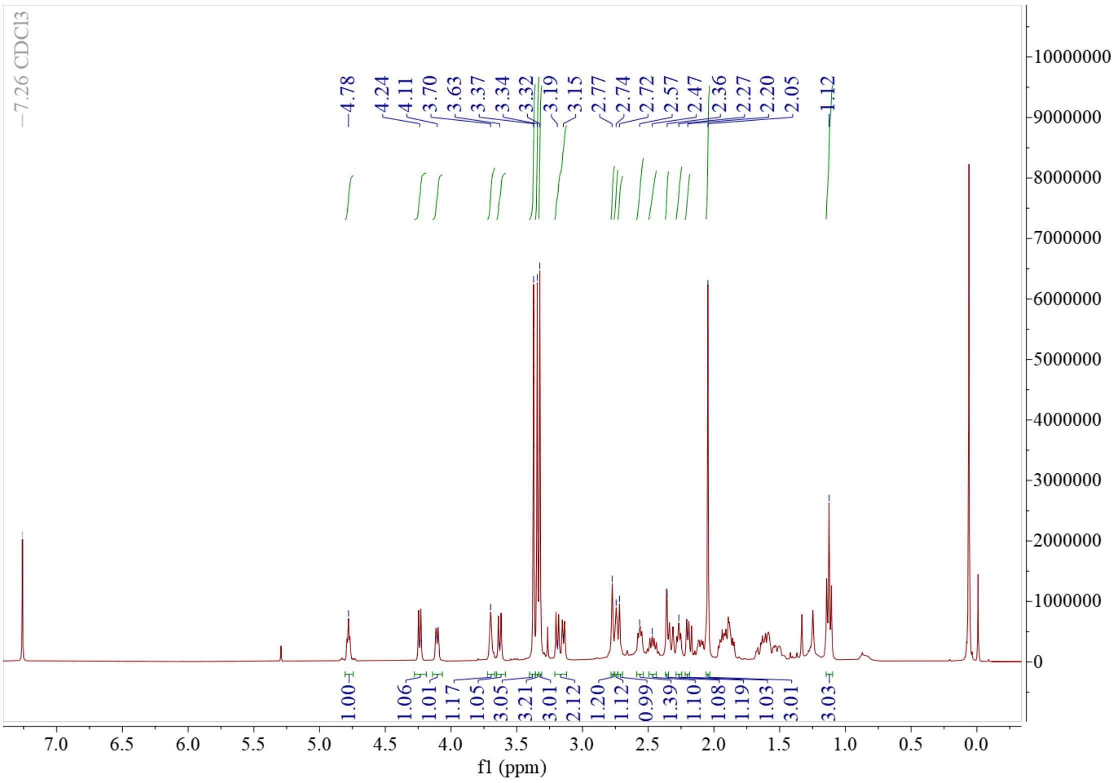

Figure S19. ^{1}H NMR (400 MHz) spectrum of compound **7** in $CDCl_3$

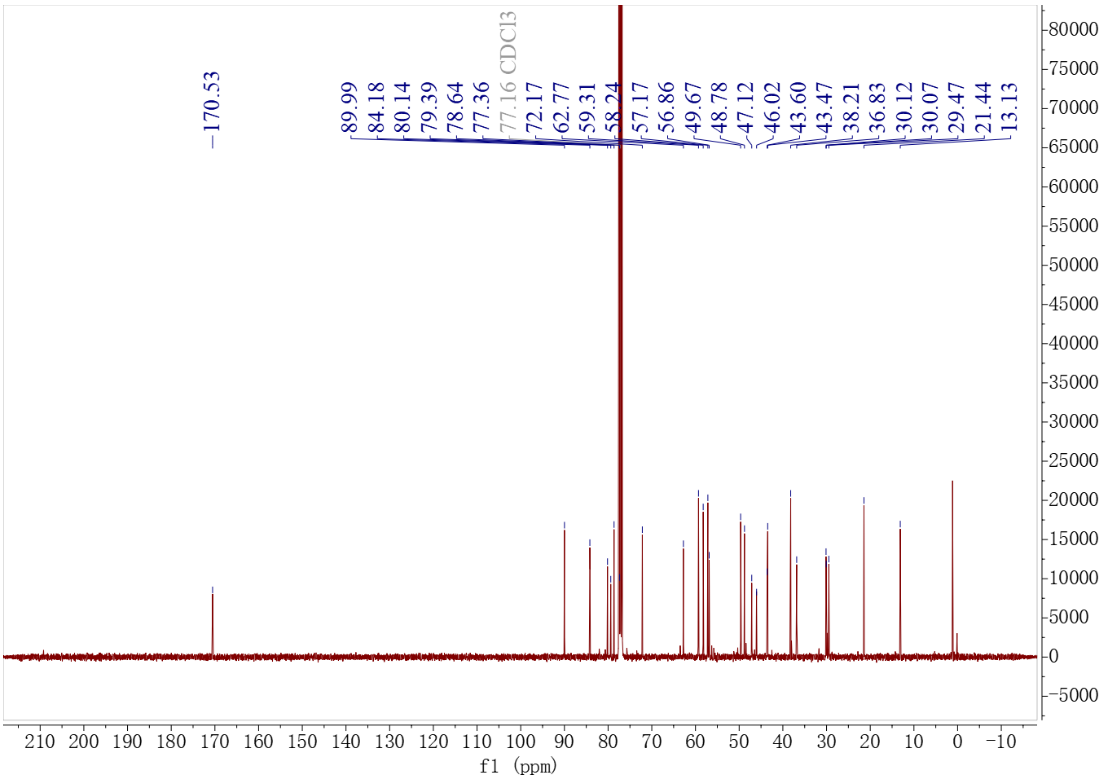

Figure S20. ^{13}C NMR (101 MHz) spectrum of compound **7** in $CDCl_3$

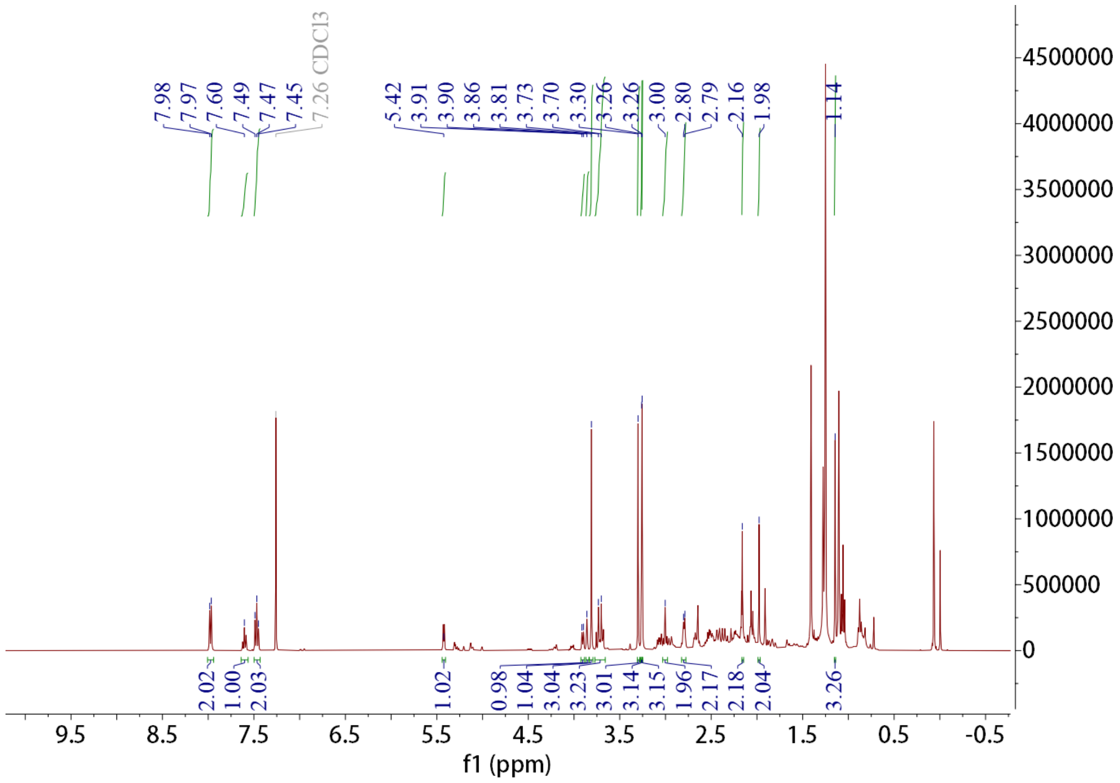

Figure S21. ^{1}H NMR (400 MHz) spectrum of compound **8** in $CDCl_3$

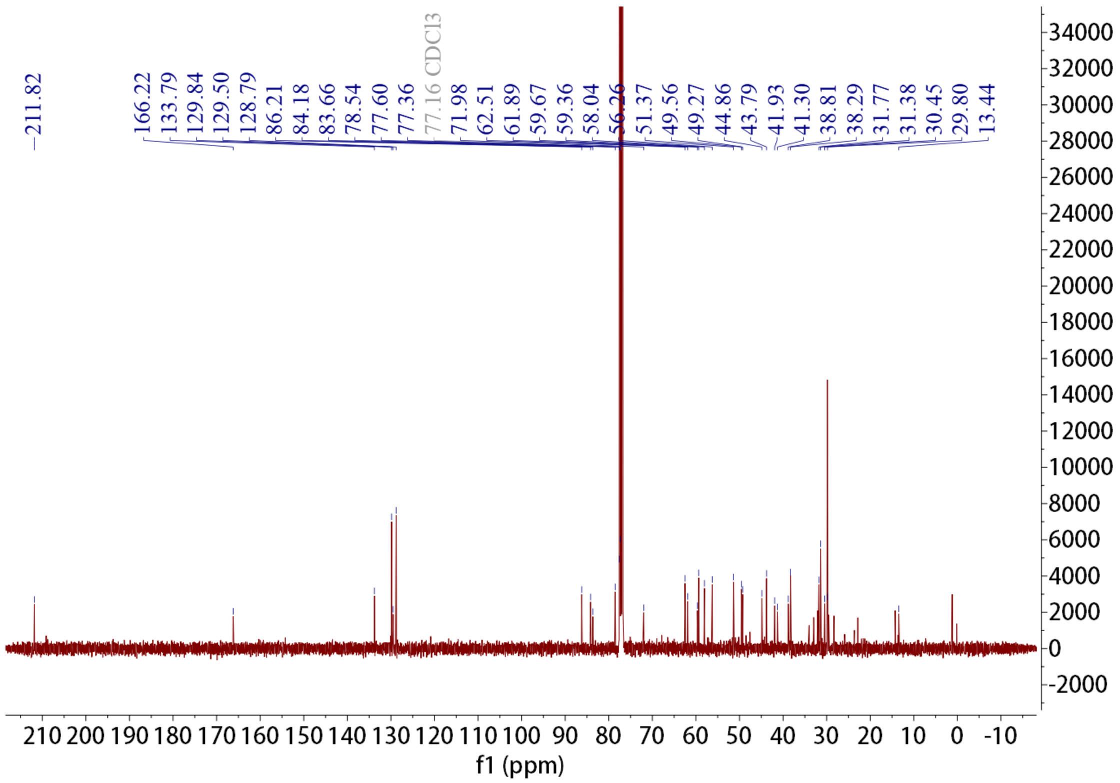

Figure S22. ^{13}C NMR (101 MHz) spectrum of compound **8** in $CDCl_3$

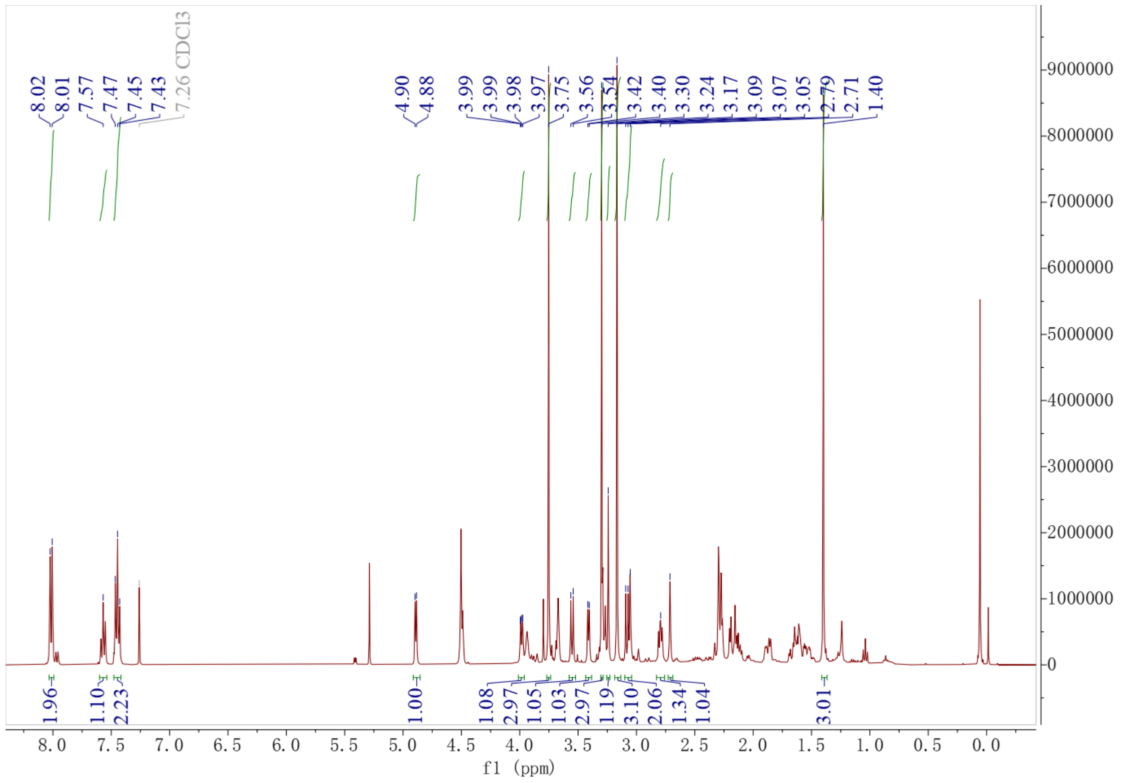

Figure S23. ^{1}H NMR (400 MHz) spectrum of compound **9** in $CDCl_3$

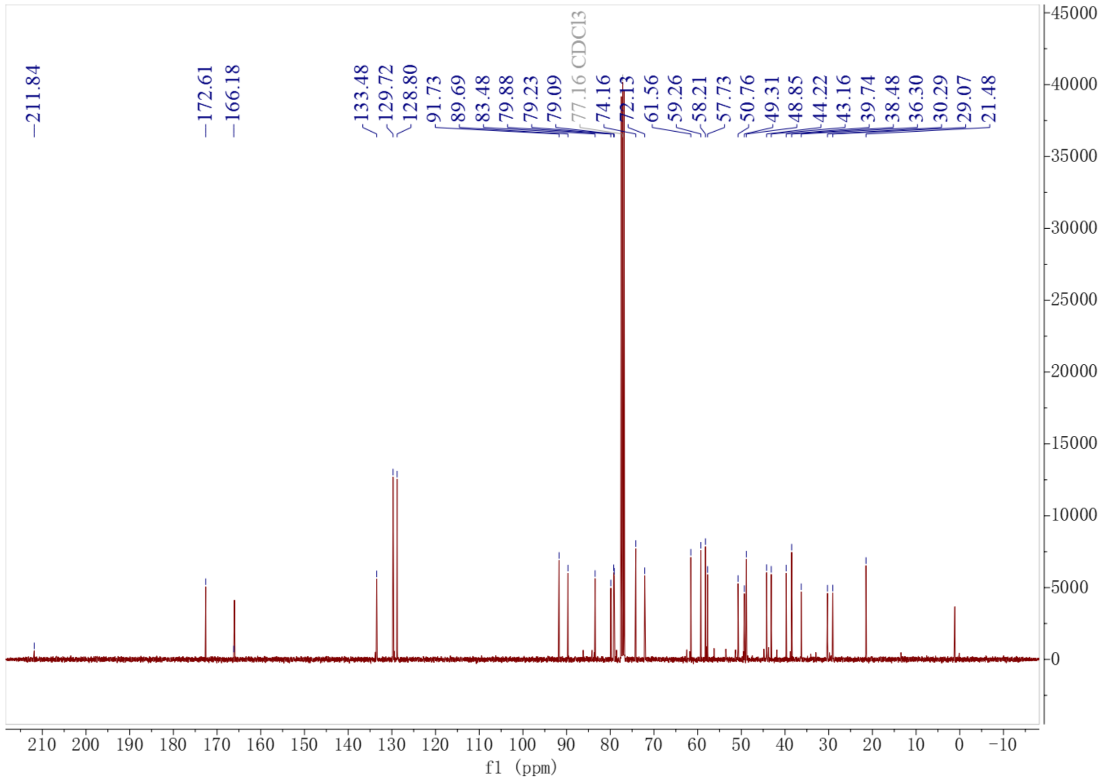

Figure S24. ^{13}C NMR (101 MHz) spectrum of compound **9** in $CDCl_3$

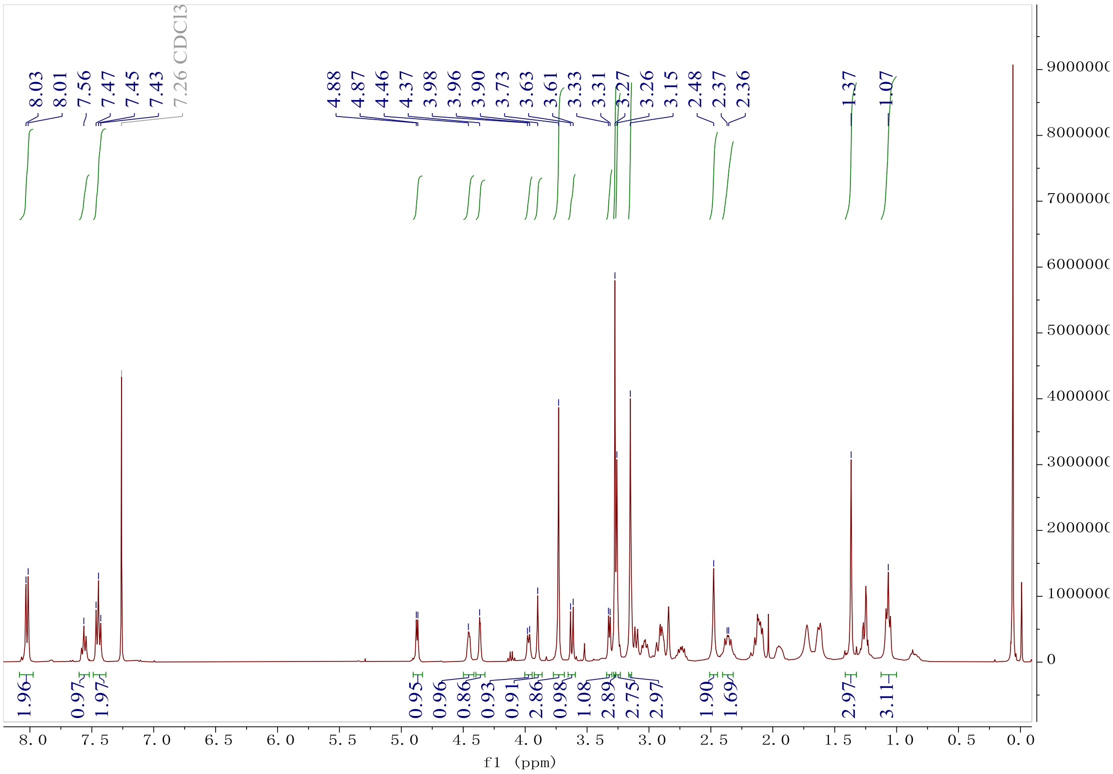

Figure S25. ^{1}H NMR (400 MHz) spectrum of compound **10** in $CDCl_3$

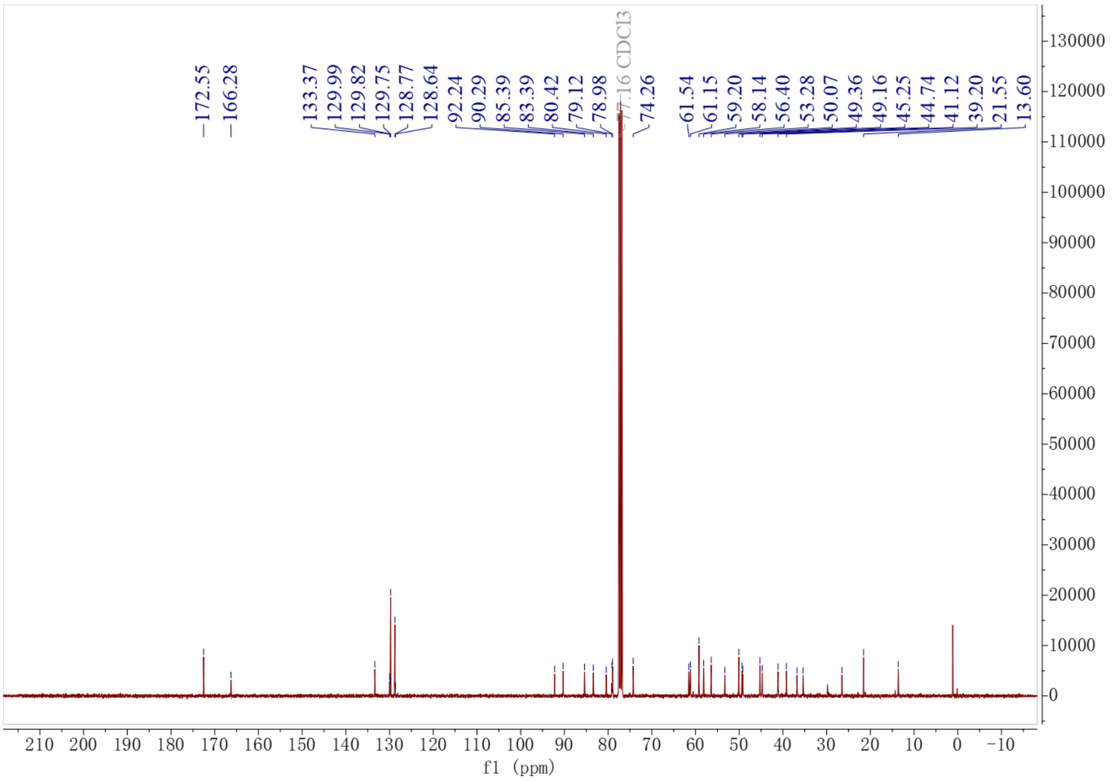

Figure S26. ^{13}C NMR (101 MHz) spectrum of compound **10** in $CDCl_3$

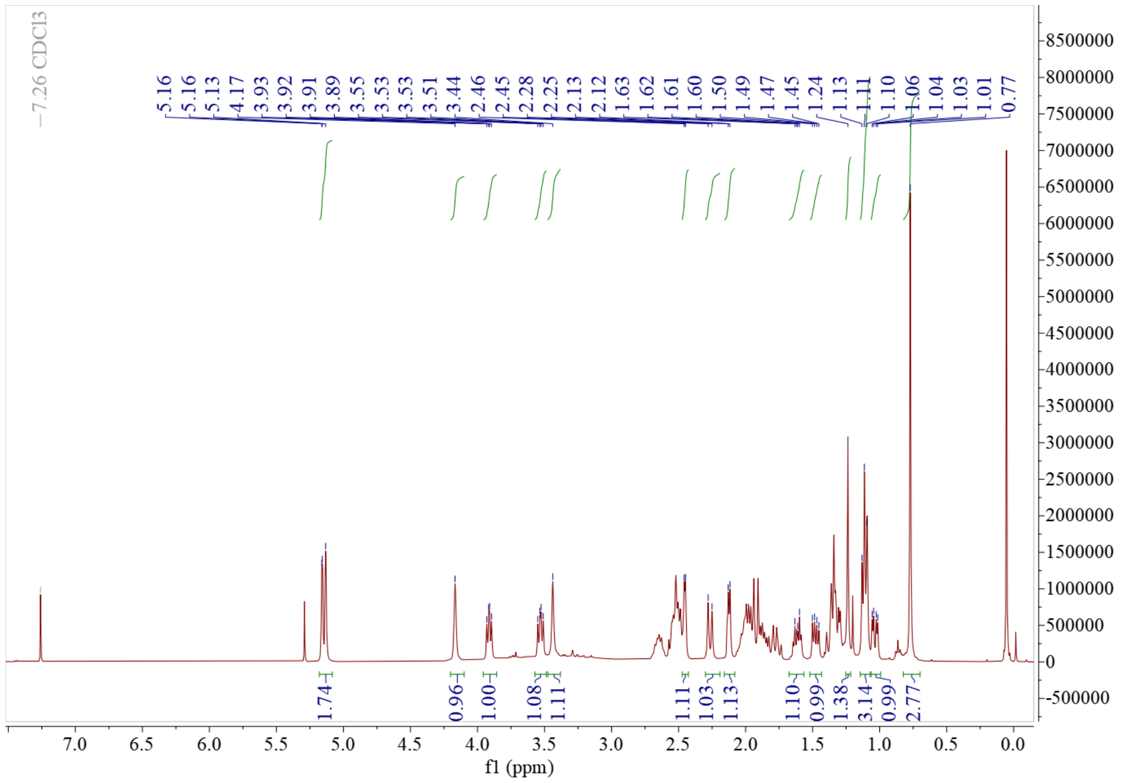

Figure S27. ^{1}H NMR (400 MHz) spectrum of compound **11** in $CDCl_3$

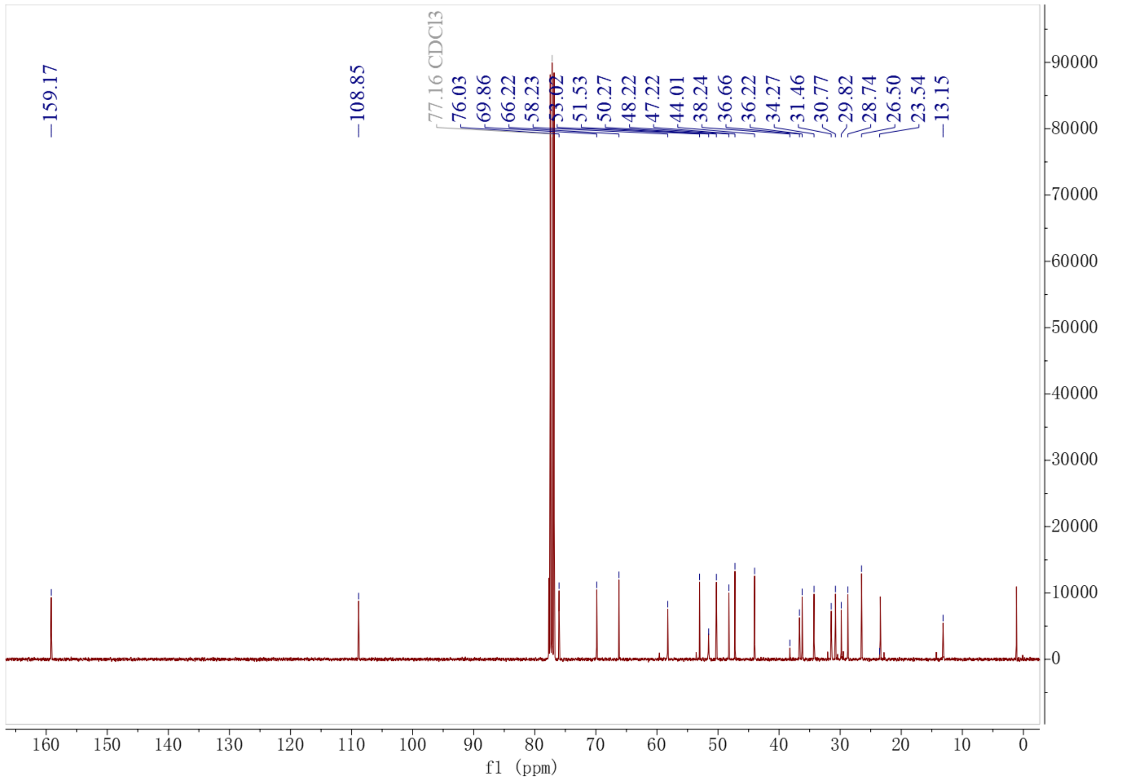

Figure S28. ^{13}C NMR (101 MHz) spectrum of compound **11** in $CDCl_3$
